# Splanchnic and Pelvic Spinal Afferent Pathways Relay Sensory Information From the Mouse Colorectum Into Distinct Brainstem Circuits

**DOI:** 10.1111/jnc.70211

**Published:** 2025-09-01

**Authors:** QingQing Wang, Alice E. McGovern, Melinda Kyloh, Grigori Rychkov, Nick J. Spencer, Stuart B. Mazzone, Stuart M. Brierley, Andrea M. Harrington

**Affiliations:** ^1^ Visceral Pain Research Group, Hopwood Centre for Neurobiology, Lifelong Health Theme, South Australian Health and Medical Research Institute (SAHMRI) Adelaide South Australia Australia; ^2^ College of Medicine and Public Health, Flinders Health and Medical Research Institute Flinders University Adelaide South Australia Australia; ^3^ Department of Anatomy and Physiology The University of Melbourne Parkville Victoria Australia; ^4^ Visceral Neurophysiology Laboratory, College of Medicine and Public Health, Flinders Health and Medical Research Institute Flinders University Adelaide South Australia Australia; ^5^ School of Biomedicine, Faculty of Health and Medical Sciences University of Adelaide Adelaide South Australia Australia

**Keywords:** brainstem and pain, distal colon, rectum, spinal cord, visceral afferent

## Abstract

The distal colon and rectum (colorectum) are innervated by two distinct spinal (splanchnic and pelvic) afferent nerve pathways. This study aimed to identify where the sensory information relayed by splanchnic and pelvic afferents integrates within the brainstem. Microinjection of transneuronal viral tracer (herpes simplex virus‐1 H129 strain expressing EGFP, H129‐EGFP) into the distal colon was used to assess the brainstem structures receiving ascending input from the colorectum. H129‐EGFP+ cells were distributed in structures involved in ascending sensory relay, descending pain modulation, and autonomic regulation in the medulla from 96 h and in the pontine and caudal midbrain at 120 h after inoculation. In a separate cohort of mice, in vivo noxious colorectal distension (CRD) followed by brainstem immunolabeling for phosphorylated MAP kinase ERK 1/2 (pERK) determined neurons activated by CRD. Many of the structures containing H129‐EGFP+ labeling also contained pERK‐labeled neurons, indicating H129‐EGFP+ labeling in colorectal signaling pathways. Surgical removal of dorsal root ganglia (DRG) containing the cell bodies of splanchnic colorectal afferent neurons significantly reduced CRD‐evoked pERK neuronal activation within the caudal ventrolateral medulla, rostral ventromedial medulla, and the lateral parabrachial nuclei. Surgical removal of the DRG containing the cell bodies of pelvic colorectal afferent neurons significantly reduced CRD‐evoked pERK neuronal activation within the rostral ventromedial medulla, lateral parabrachial nuclei, the locus coeruleus, Barrington's nucleus, and periaqueductal gray. Collectively, this study showed that the two spinal afferent pathways innervating the colorectum relay information into different brainstem structures and provide new insight into their unique roles in relaying information into the gut‐brain axis controlling colorectal sensory‐motor function.

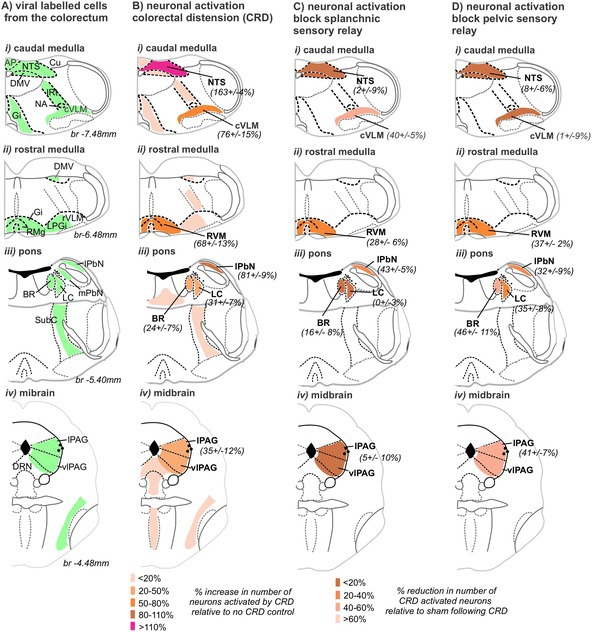

## Graphical abstract

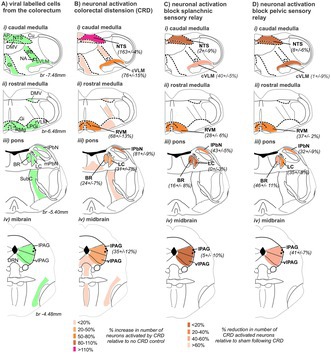

This study used transneuronal viral tracer to identify the spinal cord and brainstem circuits connected to the colorectum in the mouse (A). To establish if the regions of the brainstem labelled from the colon were functionally relevant to colorectal sensory‐motor processing, the distribution of neurons activated by distending the colon was mapped (B) and compared to viral labelling. The effect of blocking colorectal sensory relay via either the splanchnic or pelvic spinal afferent pathways on colorectal evoked neuronal activation in the brainstem was then compared (C and D). This identified how each pathway contributes to the relay of colorectal sensory information into unique brainstem circuits involved in pain relay, descending pain modulation and autonomic motor regulation.

**Abbreviations**
4 V: fourth ventricle
5‐HT: serotonin
AP: area postrema
BR: Barrington's nucleus
br: bregma
CRD: colorectal distension
Cu: cuneate nucleus
cVLM: caudal ventrolateral medulla
DGC: dorsal gray commissure
DMV: dorsal motor nucleus of the vagus
DRG: dorsal root ganglia
DRN: dorsal raphe nuclei
DVC: dorsal vagal complex
Gi: gigantocellular reticular nucleus
HSV1 H129‐EGFP: herpes simplex virus 1, H129 strain, expressing green fluorescent protein
ici: intercalated nuclei
IML: intermediolateral nuclei
lPAG: lateral periaqueductal gray
IR: immunoreactive
IRt: intermediate reticular nucleus
KF: Kölliker‐Fuse nucleus
LC: locus coeruleus
LI: lamina I
LII: lamina II
LIII: lamina III
LIV: lamina 4
lPbN: lateral parabrachial nuclei
LPgi: lateral paragigantocellular nucleus
LPGi: lateral paragigantocellular nucleus
LRt: lateral reticular nucleus
LS: lumbosacral
LSN: lateral spinal nucleus
LV: lamina 5
mPbN: medial parabrachial nuclei
NA: nucleus ambiguus
NTS: nucleus of the solitary tract
PAG: periaqueductal gray
pERK: phosphorylated MAP kinase ERK1/2
RMg: raphe magnus nucleus
ROb: raphe obscurus nucleus
RPa: raphe pallidus nucleus
RRID: Research Resource Identifier (see http://scicrunch.org)
rVLM: rostral ventrolateral medulla
rVLM: rostroventrolateral reticular nucleus
RVM: rostral ventromedial medulla
scp: superior cerebellar peduncle
SPN: sacral parasympathetic nuclei
SubC: subcoeruleus
TH: tyrosine hydroxylase
TL: thoracolumbar
vlPAG: ventrolateral periaqueductal grey

## 1 Introduction

Sensory information from the distal colon and the rectum (colorectum) is conveyed into the brain primarily via the spinal cord (Westlund 2000; Sikandar and Dickenson 2012; Kyloh et al. 2022; Harrington 2023). The colorectum is innervated by two spinal sensory afferent nerves, the lumbar splanchnic and the sacral pelvic nerves, that relay information into two segments of the spinal cord (Brierley et al. 2018; Wang et al. 2024). The splanchnic nerve relays information into the thoracolumbar (TL, T10‐L1) spinal cord, while the pelvic nerve projects into the lumbosacral (LS, L5‐S1) spinal cord (Harrington et al. 2019; Wang et al. 2024). It is well established that colorectal splanchnic and pelvic afferent fibers possess different peripheral properties that determine their activation profiles and what stimuli they respond to (Brierley et al. 2018). A large proportion of colorectal splanchnic afferent fibers are high‐threshold nociceptors, while pelvic afferent fibers are more functionally diverse (Brierley et al. 2004). Relative to the wealth of knowledge of their peripheral properties, knowledge of the spinal cord‐brain axis into which colorectal spinal afferent nerves relay to affect appropriate sensory‐motor responses is limited. Our recent studies in mice show that the dorsal horn circuits within the TL spinal cord receiving input from splanchnic colorectal afferent fibers are principally activated by noxious pressures of colorectal stretch (distension) and possess characteristics relevant to the relay of nociceptive information into the brain (Harrington et al. 2019; Wang et al. 2024). In contrast, the dorsal horn circuits in the LS spinal cord that receive input from pelvic colorectal afferent fibers are activated by innocuous and noxious pressures of colorectal distension (CRD) and demonstrate characteristics relevant to nociceptive and homeostatic interoceptive relay to the brain, as well as sensory integration into autonomic motor circuits (Harrington et al. 2019; Wang et al. 2024). Expanding on these findings, this study aimed to determine how the differences between splanchnic and pelvic colorectal afferent pathways are reflected further along the spinal cord‐brain axis by assessing their influence on where colorectal processing occurs within the brainstem.

The brainstem is the first site within the brain where viscerosensory information is integrated to support the behavioral and affective sensory‐motor responses associated with visceral nociception (Westlund 2000). Sensory‐motor responses evoked by noxious CRD are attenuated by decerebration at the level of the brainstem pontine but not rostral to the brainstem midbrain (Ness and Gebhart 1988). Numerous brainstem structures regulate how colorectal sensory‐motor information is relayed into cortico‐limbic structures relevant to pain perception and discrimination, as well as being functionally relevant to descending pain modulation and autonomic responses (Monnikes et al. 1994, 2003; Pavcovich et al. 1998; Naitou et al. 2018; Nakamori et al. 2018, 2019; Lyubashina et al. 2019, 2022). Studies in rats using transneuronal tracing with pseudorabies virus (PRV) have identified the particular brainstem structures synaptically connected to the colorectum (Pavcovich et al. 1998; Valentino et al. 2000; Vizzard et al. 2000; Rouzade‐Dominguez et al. 2003; He et al. 2018). Demonstrating the connectivity of these structures to the colorectum via the spinal cord, spinal cord transection (at T8) reduces PRV‐labelling from the colon in the medulla ventrolateral raphe, pons (A7), Barrington's nucleus (BR), locus coeruleus (LC), parabrachial nucleus (PbN), and midbrain (periaqueductal gray (PAG) and red nucleus) (Vizzard et al. 2000). Many of these structures receive direct input from the sensory and autonomic pathways ascending through the spinal cord, as well as being part of interconnected brainstem networks with descending output to the spinal cord (Parker et al. 2020). The contribution of signaling within the spinal afferent pathways, ascending via either the TL (splanchnic) or LS (pelvic) spinal cord, to colorectal processing within these brainstem sensory‐motor networks remains to be established.

To address such knowledge gaps, our first aim was to identify the brainstem structures connected to the colorectum via spinal ascending pathways in the mouse. To do this, we tracked the transneuronal transport of a herpes simplex virus type 1 strain (H129) expressing EGFP (H129‐EGFP) within the spinal pathways and brainstem after its injection into the colorectal wall. HSV‐1 H129 has been shown to have a preferential, but not exclusive, anterograde direction of transport (Rinaman and Schwartz 2004; McGovern, Davis‐Poynter, Farrell, and Mazzone 2012; McGovern, Davis‐Poynter, Rakoczy, et al. 2012; Wojaczynski et al. 2015). This contrasts with PRV, which is described as having a preferential retrograde direction of transport through central circuits (Vizzard et al. 2000; Parker et al. 2020). To target the spinal pathways, rather than the vagal afferent pathways, H129‐EGFP injections were made into the region of the colorectum that we have shown previously to be densely innervated by splanchnic and pelvic spinal afferent fibers but sparsely innervated by vagal afferent fibers (Wang et al. 2024). In vivo noxious CRD followed by immunolabeling for the neuronal activation marker phosphorylated MAP kinase ERK 1/2 (pERK) was then used to map the distribution of brainstem nuclei functionally relevant to colorectal sensory‐motor processing and assess how this distribution aligns with where H129‐EGFP positive labeling was observed. We then aimed to determine the relative contribution of splanchnic and pelvic afferent pathways to ascending sensory signaling by assessing CRD‐evoked neuronal activation in brainstem structures relevant to sensory relay, descending pain modulation, and autonomic outflow. To differentiate contributions from splanchnic and pelvic pathways, we used a dorsal root ganglion (DRG) removal model (Kyloh et al. 2022). We have shown previously that visceromotor responses evoked from rectal distension, at pressures in the physiological and noxious ranges, are attenuated by removing LS DRG (L5‐S1), which contains cell bodies of colorectal pelvic afferents. Visceromotor responses evoked by noxious distension of the distal colon were attenuated by removing the TL DRG (T13‐L1), which contain the cell bodies of colorectal splanchnic afferents. The outcomes of these assessments provided novel information on the relative contribution that the splanchnic and pelvic spinal afferent pathways have in shaping colorectal processing within brainstem sensory‐motor circuits.

## 2 Materials and Methods

### 2.1 Animals

Female and male C57BL/6J (8–16 weeks, weight range 18–30 g) mice were used for all experiments. A total of 59 mice were used. All animal procedures were performed in accordance with the approval of Animal Ethics Committees of the South Australian Health and Medical Research Institute (SAHMRI; Application SAM190), the University of Melbourne (Application 1714311.1) and Flinders University (Approval no. 861–13). The HSV1 H129‐EGFP transneuronal tracing and pERK neuronal activation studies were performed in separate cohorts of mice. Mice used for HSV1 H129‐EGFP transneuronal tracing studies were sourced from the Animal Resources Centre (ARC) (Perth, Australia). Mice for pERK neuronal studies were sourced from in‐house colonies of the SAHMRI Bioresources Facility (a specific and opportunistic pathogen‐free facility), and mice from the School of Medicine Animal House facility (SoMAF), Flinders University, were used for DRG removal studies. In all locations, mice were group‐housed (3–5 mice per cage) prior to surgical approaches and then individually housed after procedures within ventilated cages (IVC), stored on IVC racks within a temperature‐controlled environment of 22°C and a 12 h light/12 h dark cycle. Mice from SAHMRI were housed on dust‐free coarse chip aspen bedding (cat# ASPJMAEBCA, PuraBed, Niederglatt, Switzerland), mice from SoMAF were housed on corn cob bedding (cat# ASCCB‐C, PuraCob, Able Scientific, SA, Australia), and mice from the University of Melbourne were housed on dust‐free absorbent paper pellets. In each location, mice had free access to autoclaved reverse osmosis water and rat and mouse chow (SAHMRI: LabDiet JL Auto6F Rat and Mouse chow, cat#5 K52, St. Louis, MO; USA; SoMAF and University of Melbourne: SF00‐100 Irradiated Rat & Mouse chow, Specialty Feeds Pty Ltd., WA, Australia). Animals were assigned to different experimental groups arbitrarily.

### 2.2 HSV1 H129‐EGFP Transneuronal Tracing From the Colorectal Wall

Anterograde transneuronal tracing was performed in female and male mice from the colorectum using a herpes simplex virus 1, H129 strain, expressing EGFP (HSV1 H129‐EGFP). The wildtype HSV‐1 H129 source origin, construction of the H129‐EGFP recombinant virus, and its validation for preferential trans‐synaptic movement in the anterograde direction to label the central circuitry arising from peripheral structures have previously been described (Barnett et al. 1995; Rinaman and Schwartz 2004; McGovern, Davis‐Poynter, Farrell, and Mazzone 2012; McGovern, Davis‐Poynter, Rakoczy, et al. 2012; McGovern et al. 2015; Wojaczynski et al. 2015). Mice underwent anesthesia induction using inhalation isoflurane at 4% in 1 L/min oxygen, which was then maintained at 1.5%–2% in 1 L/min oxygen. A small aseptic abdominal incision was then made, and the colorectum was located. Aliquots of viral stocks were thawed from −80°C, and 10 μL of 7 × 10^7^ pfu/mL of H129‐EGFP virus was injected into the subserosa‐musculature wall of the colorectum (covering 1 cm proximal to the pelvic bone through to immediately below the pelvic bone). Within this region, 3–6 injections (1–2 μL/injection) were made into the colorectal wall at bilateral and midline locations (Figure S1) using a 30‐gauge needle (cat#HAMC7803‐07, point style: 4 (10°–12°); Hamilton Company, Bio‐Strategy, Campbellfield, Vic, Australia) attached to a Hamilton 5 μL syringe (cat#HAMC7634‐01 5 μL 700 series RN syringe; Hamilton Company, Bio‐Strategy). The needle tip was inserted into the subserosal wall space and tunneled a short distance caudally, ensuring the needle tip was always visible within the wall. The viral solution was expelled as the needle was gradually pulled out of the needle track. Care was taken to minimize leakage of the virus during injections into the colon wall by using cotton‐tip applicators at injection sites. The abdominal incision was then sutured closed, and prior to withdrawal of anesthesia, animals were administered a dose of analgesia (buprenorphine, 0.5 mg/kg) via subcutaneous injection. Mice were then group‐housed and monitored twice daily for signs of weight loss, altered colonic motility (changes to fecal output and form), and stereotypical behavior (altered gait or locomotion, reduced or excessive grooming, lack of nesting, and repetitive movements). Mice showed no clinical signs 24–48 h post‐viral tracing and in varying severity from 72 h through to 120 h post‐inoculation. Mice underwent transcardial perfusion fixation at arbitrarily designated survival points or upon accumulative or severe clinical signs outlined above. Tissue (colon, DRG, spinal cord, and brain) was collected 24 h post‐inoculation (*N* = 3 mice inoculated, 2F:1M), 48 h post‐inoculation (*N* = 4 mice inoculated, 2F:2M), 72 h post‐inoculation (*N* = 4 mice inoculated, 2F:2M), 96 h post‐inoculation (*N* = 5 mice inoculated, 3F:2M), and 120 h post‐inoculation (*N* = 8 mice inoculated, 7F:1M). Survival times post‐inoculation were selected based on pilot studies and presentation of clinical signs based on previous studies in rats mapping the time course of H12‐EGFP infection from the trachea (McGovern, Davis‐Poynter, Farrell, and Mazzone 2012; McGovern, Davis‐Poynter, Rakoczy, et al. 2012). 72 h post‐infection of the trachea, H129‐EGFP‐labeling was visible in vagal sensory ganglia, and by 96 h post‐infection, H129‐EGFP‐labelling was visible in the thalamus. Similar survival times of 72‐, 96‐, 120 h post‐H129 inoculation of the rat stomach have been used to show H129 labeling in vagal sensory ganglia 72 h post‐inoculation (earlier times not reported on) and in the thoracic spinal cord and medulla 96 h after post‐inoculation and widespread in the brainstem and diencephalon 120 h post‐infection (Rinaman and Schwartz 2004). A survival time of 5 days post‐inoculation of the rat small intestine has been used to assess H129 labeling throughout the brain (Parker et al. 2020).

### 2.3 In Vivo CRD

Mice (*N* = 10 mice, 5F:5M), separate from those used for HSV1 H129‐EGFP transneuronal tracing, underwent in vivo CRD to identify neurons activated by colorectal input. Mice underwent anesthesia induction using inhalation isoflurane at 5% in 1 L/min oxygen and maintained at 3% in 1 L/min oxygen, during which mice were given an enema of sterile saline followed by insertion of a 2 cm balloon catheter into the perianal canal. When the base of the balloon was localized approximately 0.2 cm proximal to the anal verge, the catheter tube was secured in place by taping it to the base of the tail (Christianson and Gebhart 2007). Four minutes after balloon placement, mice were removed from anesthesia, immediately placed in a Perspex box, and the balloon catheter was distended to a pressure of 80 mmHg via a syringe attached to a three‐way tap and monitored via a sphygmomanometer. Distension was held for 10 s, then released for 5 s. This sequence was repeated 5 times and completed prior to mice regaining full consciousness (Harrington et al. 2019; Wang et al. 2024). Immediately after the fifth distension, mice were placed back into the isoflurane induction chamber (as conditions above) and, within 30 to 40 s, given an overdose of euthanasia agent (via intraperitoneal injection, 60 mg/kg pentobarbitone sodium solution, Lethabarb, Virbac Australia, Milperra, NSW, Australia) and underwent transcardial perfusion fixation. Fixation was completed within 5to 6 min after the final distension. A separate group of mice (*N* = 10 mice, 5F:5M) underwent the same procedure as above for balloon insertion but did not have the balloon distended (no CRD) when placed in the Perspex box for 1 min.

### 2.4 Surgical Removal of TL and LS DRG

In order to compare how signaling via the splanchnic or pelvic afferent pathways shapes colorectal evoked neuronal activation in the brainstem, a separate cohort of female mice underwent surgical removal of DRG at either vertebrae levels T13–L1 (thoracolumbar DRG TL removed) or L5–S1 (lumbosacral DRG LS removed) (Kyloh et al. 2022) or sham surgery 8–9 days prior to in vivo CRD procedure described above (*N* = 5 mice/group). Mice underwent anesthesia induction using inhalation isoflurane at 4% in 1 L/min oxygen, which was then maintained at 1.5%–2% in 1 L/min oxygen. Animals were positioned on a thermostat‐controlled heat mat to maintain body temperature throughout the procedure (Adloheat, Pakenham, Vic, Australia). Before incision, animals were administered a dose of analgesia via subcutaneous injection (buprenorphine, 0.5 mg/kg (Temvet)). The dorsal surface was shaved and cleaned with 0.5% chlorhexidine and a 70% alcohol swab (Briemar). An incision (~20 mm in length) was made along the dorsal midline, and skeletal muscles were retracted to expose the vertebral column. A laminotomy was performed to remove small fragments of vertebral bone from the dorsal (uppermost) surface of each DRG to expose the ganglion but not the dura or spinal cord. The dorsal nerve root between DRG located at both sides of the vertebrae at levels T13–L1 (thoracolumbar DRG TL removed) and L5, L6, and S1 (lumbosacral DRG LS removed) was severed on either side of the ganglion, and the ganglion was removed entirely (Kyloh et al. 2022). Sham experimental groups underwent laminotomy and the DRG exposure but no DRG removal surgery. Following removal of DRG or sham surgery, the wound was irrigated with 0.5% Bupivicaine (Marcain, AstraZeneca), and the muscle was closed with individual 5.0 polyglycolic acid absorbable sutures (Silverglide). Skin was closed with 6.0 Nylon non‐absorbable suture (Silverglide), and the site was cleaned with 0.5% chlorhexidine and a 70% alcohol swab. Prior to withdrawal of anesthesia, animals were administered a second subcutaneous dose of 0.5 mg/kg buprenorphine (Temvet) and subcutaneous antibiotics, 100 mg/kg ampicillin (Alphapharm) and 10 mg/kg Baytril (Bayer). Following withdrawal of anesthesia, animals recovered on a heat mat until fully mobile, and then they were returned to their home cage. Postoperatively, animals received 0.1 mg/kg oral buprenorphine (Schering Plow) in Nutella (Ferrero) paste at 24‐h intervals for 72 h.

### 2.5 Transcardial Perfuse Fixation and Tissue Processing

Mice were euthanized with an overdose of Lethabarb, via intraperitoneal injection, prior to opening the chest cavity, and 0.5 mL heparinized‐saline (50 IU/5 mL, Pfizer) was then injected into the left ventricle, followed by insertion of a 22‐gauge needle attached to a peristaltic perfusion pump. The right atrium was then snipped, allowing for perfusate drainage. Warm 0.1 M phosphate buffer 0.08 M NaH2PO4 (cat#SA061, ChemSupply Australia), 0.02 M Na2HPO4 (cat#567547, EMD Millipore, MA, USA) was perfused prior to ice‐cold 4% paraformaldehyde (cat#158127, Sigma‐Aldrich, MO, USA) in 0.1 M phosphate. Following complete perfusion of the spinal cord and brain, and in the case of HSV1‐H129‐EGFP‐inoculated mice, the distal colon and the thoracolumbar T10‐L1 and lumbosacral L5‐S1 DRG were collected (Robinson et al. 2004; Christianson et al. 2006). The lowest rib was used as an anatomical marker of T13 DRG. The spinal cord levels T10‐L1 were identified by DRG root insertion points or vertebra level, and the spinal cord from below vertebra level L2 was removed, which contains spinal cord levels L5‐S1. The proximal edge of the distal colon was marked by opening the circumference. All tissue was post‐fixed in 4% paraformaldehyde in 0.1 M phosphate buffer at 4°C for 18 to 20 h and then cryoprotected in 30% sucrose (cat# S9378, Sigma‐Aldrich) in 0.1 M phosphate buffer overnight at 4°C. Ganglia were then placed in 100% OCT (cat# 4583, Tissue‐Tek O.C.T. Compound, Sakura Finetek, Netherlands) and snap‐frozen in liquid nitrogen. The spinal cord and brain underwent an additional 24‐h incubation at 4°C in 50% OCT/30% sucrose/phosphate buffer solution before freezing in 100% OCT. Tissue was cryosectioned, and sections were placed onto gelatin‐coated slides for visualization of H129‐EGFP‐labelling (colon, DRG, spinal cord, and brain 20–50 μm sections) or for immunolabeling (spinal cord and brain 10 μm thick sections). Sections for H129‐EGFP‐labelling visualization were air dried for an hour before being washed in 0.2% Triton‐X 100 (cat# X‐100, Sigma‐Aldrich, MO, USA) in 0.1 M phosphate‐buffered saline before coverslipping with ProLong Diamond Antifade Mountant with DAPI (cat# P36966; Invitrogen, ThermoFisher Scientific). For immunolabeling, two slides (100 μm apart) covering medulla, pons, and midbrain levels were randomly selected per mouse. The spinal cord and brain levels were identified ex vivo using the Allen Institute Mouse Spinal Cord Atlas (https://mousespinal.brain‐map.org/imageseries/showref.html) (Lein et al. 2007) and the Paxinos and Franklin Mouse Brain Atlas (Paxinos and Franklin 2001).

### 2.6 pERK Immunolabeling

Immunolabeling for pERK using HRP‐DAB detection (EnVision FLEX TRS low pH Mini Kit reagents cat#s K8805, X090, S0809, and GV823, Agilent Dako, Agilent Technologies Australia, Mulgrave, Australia) and the DAKO Omnis auto‐stainer (Agilent Technologies), followed by hematoxylin staining, was performed as previously described (Wang et al. 2024) using the primary antibody Rabbit anti‐pERK ½ (cat# MAB4370, Cell Signaling Technology, Genesearch, Qld). Rabbit monoclonal antibody detects endogenous levels of p44 and p42 MAP Kinase (Erk1 and Erk2) when dually phosphorylated at Thr202 and Tyr204 of Erk1 (Thr185 and Tyr187 of Erk2) and singly phosphorylated at Thr202. The antibody does not cross‐react with the corresponding phosphorylated residues of either JNK/SAPK or p38 MAP kinases (manufacturer's specifications) and binds specifically to a 44 kDa band in stimulated mouse tissue (Miyaji et al. 2009).

### 2.7 pERK and Neurochemical Marker Immunolabeling

Immunofluorescence for pERK and neurochemical markers was performed as previously described (Harrington et al. 2019; Wang et al. 2024). Briefly, after air drying for 1 h, sections were washed with 0.5% Triton‐TX 100 in 0.1 M phosphate‐buffered saline (T‐PBS) to remove excess OCT. Non‐specific binding of secondary antibodies was blocked using either 5% normal chicken serum (cat# CHBX0010; Applied Biological Products, SA, Australia) or 5% normal goat serum (cat# GTBX0050; Applied Biological Products) diluted in T‐PBS for 30 min at room temperature. Tissue sections were then incubated with primary antisera mouse anti‐tyrosine hydroxylase (TH; Isotype IgG1; 1:1000, cat #: 22941, RRID: AB 572 268, ImmunoStar Inc., Hudson, WI, USA) or goat anti‐serotonin (5HT; 1:1000, cat #: 20079, RRID: AB_572262, ImmunoStar Inc) for 24 h at room temperature, diluted in T‐PBS. Sections were then washed in T‐PBS and incubated for 1 h at room temperature with the appropriate secondary antibody conjugated, either chicken anti‐rabbit IgG1 AF488 (1:200, cat# A‐21441, RRID: AB_2535859), Chicken anti‐mouse IgG1 AF594 (1:200, cat# A‐21125, RRID: AB_141593, ThermoFisher Scientific), or chicken anti‐goat AF594 (1:200, cat# A‐21468, RRID: AB_141859, ThermoFisher Scientific). Sections were then washed in T‐PBS before mounting in ProLong Glass Antifade Mountant with NucBlue and coverslipped. Slides were allowed to dry for 24 h prior to visualization. The anti‐TH and anti‐5HT antisera have been used extensively to label regions of the mouse brain that are known to contain TH (Asmus et al. 2008; Churchill et al. 2019) and 5‐HT (Iwasaki et al. 2018; Hingorani et al. 2022) neuronal cell bodies.

### 2.8 Microscopy

Fluorescence was manually visualized using a confocal laser scanning microscope (Leica TCS SP8X, Germany) or an epifluorescent microscope (Olympus BX51; Olympus, Tokyo, Japan) or autoscanned using an epifluorescence ZEISS Axioscan 7 Slide Scanner (Carl Zeiss Microscopy, Germany) with a 40× air objective (EGFP in spinal cord and brainstem sections only). pERK‐DAB staining was imaged using a NanoZoomer slide scanner (Hamamatsu, Japan) with a 40× objective. Confocal images (1024 × 1024 pixels) were obtained with oil immersion 20×–63× objectives and software zoom of 2 to 4× magnification. Sequential scanning (5‐line average) was performed with the following settings using a tunable white light laser and photomultiplier detectors: 495 nm‐excitation and 503/538 nm‐emission detection for EGFP/AF488 and 561 nm‐excitation and 570–625 nm emission detection for AF594. Spinal cord and brainstem sections were optically sectioned (2 μm thick sections), and z‐projected images were reconstructed as z‐stacks (10–50 μm).

Images were processed using LAS Lite (Leica), ZEN BLUE (Carl Zeiss Microscopy), FIJI (NIH, MD, USA), and CorelDRAW Graphic Suite 2021 (Corel Corporation, CA, USA) software. Other than making moderate adjustments for contrast and brightness, the images were not manipulated in any way.

### 2.9 Quantification and Statistics

Quantification of H129‐EGFP‐labeled cells was determined from single‐plane epifluorescence images obtained from the ZEISS auto‐scanner, viewed using ZEN BLUE (Zeiss) software, from 10 sections per spinal cord level per mouse per time point and2 to 5 sections per brainstem level per mouse per time point. The density of H129‐EGFP + cells over time was expressed as scattered (1–10 H129‐EGFP cells/section), dense (11–20 H129‐EGFP cells/section), and substantial infections (21+ H129‐EGFP cells/section) (Vizzard et al. 2000; Bassi et al. 2022). Quantification of pERK‐immunoreactive neurons was performed from scanned images of pERK‐HRP immunolabeled sections, using QuPath (Queens University, Belfast, Northern Ireland) (Bankhead et al. 2017). The number of pERK neurons per section was obtained from 5 to 10 sections per/spinal cord region and 1 to 3 sections per brain level per mouse. Only cells with a neuronal morphological profile and intact nuclei (identified by NucBlue or hematoxylin counterstain) were included in the counts. At the time of slide scanning, the images produced were assigned random numbers that de‐identified their experimental group for subsequent neuronal quantification. The scanned images were then opened and viewed using NDPview2 (Hamamatsu, Japan) software or ZEN BLUE software, and the experimenter was unaware of the study groups during the counting. The data distribution was determined by the GraphPad Prism 9 normality and lognormality Shapiro–Wilk tests. The specific number of mice in each experimental group (N), the samples per section per mouse (n) and details of the tests used for statistical comparisons between experimental groups are outlined within the relevant figure legends. Unpaired *t*‐tests and one‐Way ANOVA with Bonferroni multiple comparison tests were used for parametrically distributed data, and for non‐parametric data, the one‐way Kruskal‐Wallis test with Dunn's multiple comparisons tests was used. No tests were performed for outliers. The sample sizes were based on previous studies of a similar nature (McGovern, Davis‐Poynter, Farrell, and Mazzone 2012; McGovern, Davis‐Poynter, Rakoczy, et al. 2012; McGovern et al. 2015; Kyloh et al. 2022; Wang et al. 2024). See Table S1 for a full statistical report in a stand‐alone table of analysis.

## 3 Results

### 3.1 Distribution of HSV‐1 H129‐EGFP Infected Cells (H129‐EGFP+) in the Spinal Cord and Brainstem at Different Time Points Following Colorectal Inoculation

**FIGURE 1:**
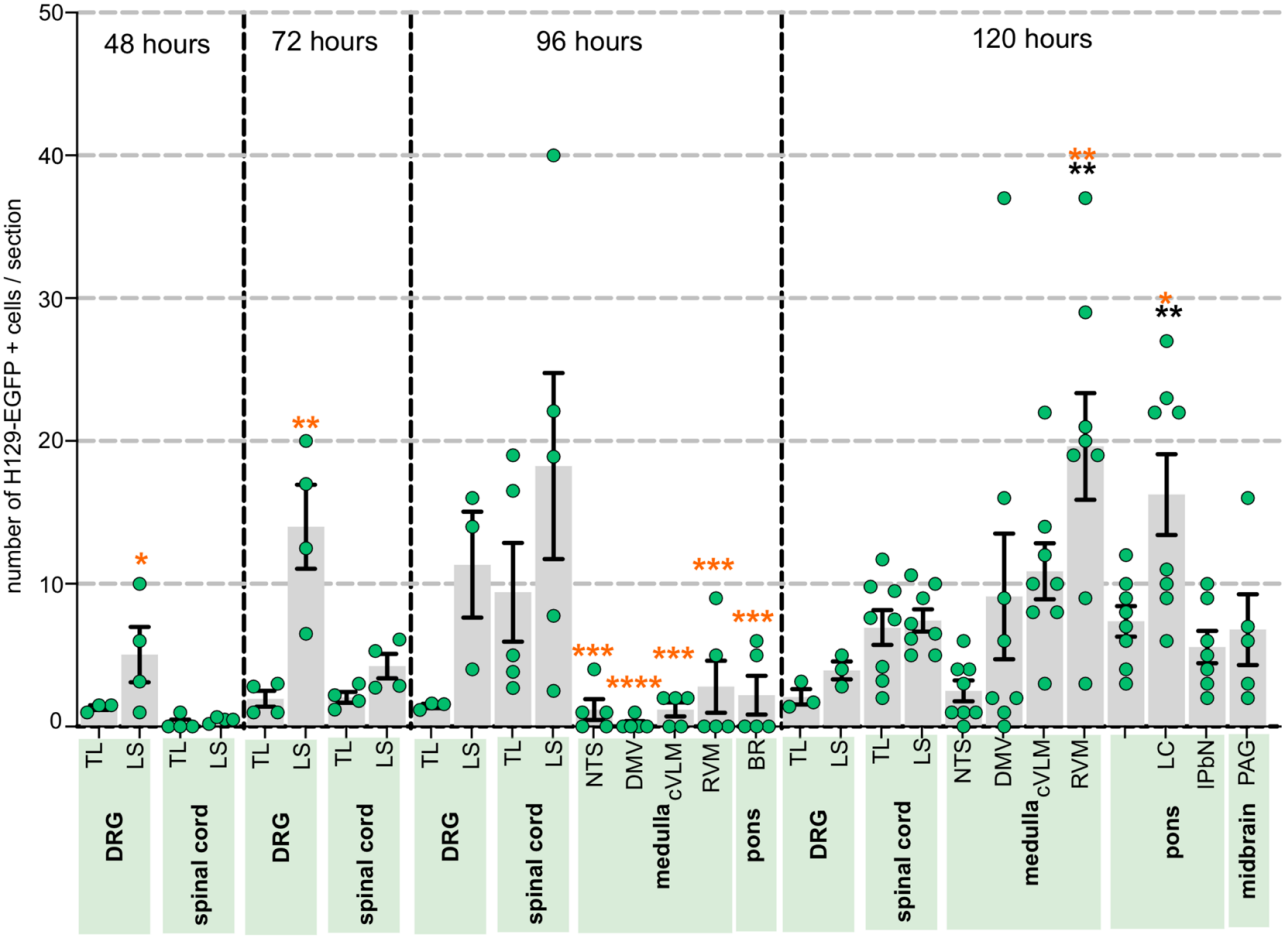
H129‐EGFP+ labelling migrated through DRG, spinal cord, and brainstem in a time‐dependent fashion following colorectal HSV‐1 H129‐EGFP inoculation. The mean number of H129‐EGFP+ cells within sections of dorsal root ganglia (thoracolumbar, TL; and lumbosacral, LS), TL and LS spinal cord dorsal horn, medulla (nucleus of the solitary tract, NTS; dorsal motor nucleus of the vagus, DMV; caudal ventrolateral medulla, cVLM; and rostral ventromedial medulla, RVM), pons (Barrington's nucleus, BR; locus coeruleus, LC; and lateral parabrachial nucleus, lPbN), and the midbrain (periaqueductal gray, PAG) in tissue collected 48‐, 72‐, 96‐, 120‐h post‐HSV‐1 H129‐EGFP colorectal inoculation. Statistical comparisons between the mean number of H‐129 EGFP+ cells in the spinal cord dorsal horn at TL (black stars) and LS (orange stars) spinal segments were made to the mean number of H‐129 EGFP+ cells in other regions, *p < 0.05, **p < 0.01, ***p < 0.001, ****p < 0.0001 determined by one‐way ANOVA (parametric data) with post hoc Bonferroni multiple comparison tests used between regions. Data points represent the mean number of H129‐EGFP+ cells/section of individual mice, gained from 2 to 10 sections/mouse collected 48 h (*N* = 4, 2F:2M), 72 h (*N* = 4 mice, 2F:2M), 96 h (*N* = 5 mice 3F:2M), and 120 h (*N* = 8 mice, 7F:1M, PAG 120 h *N* = 5 mice, 5F) post‐inoculation. Error bars represent the standard error of the mean.

**FIGURE 2:**
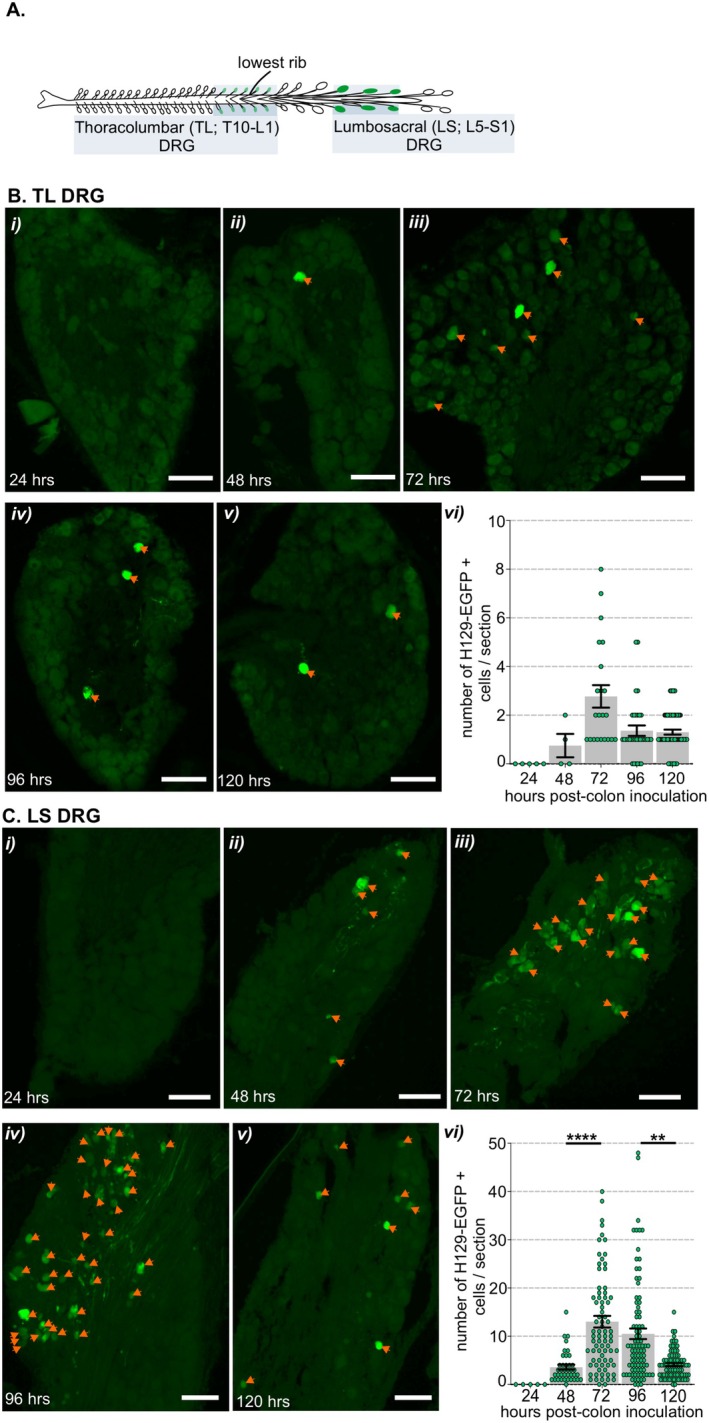
Distribution of H129‐EGFP+ cells within the thoracolumbar (TL) and lumbosacral (LS) dorsal root ganglia (DRG) following colorectal HSV‐1 H129‐EGFP inoculation. (A) Schematic showing the dorsal root ganglia (DRG) collected at spinal levels, known to contain colorectal sensory afferent neurons, thoracic 10 through to lumbar 1 (Thoracolumbar, TL. T10‐L1) and lumbar 5 through to sacral 1 (Lumbosacral, LS. L5‐S1) in which HSV‐1 H129‐EGFP‐labeling was assessed in 24‐h increments after colorectal inoculation. (B, C) Representative photomicrographs of H129‐EGFP+ cells (green, indicated by orange arrows) within sections of (B) TL DRG (T10‐L1) and (C) LS DRG (L5‐S1) at (i) 24‐, (ii) 48‐, (iii) 72‐, (iv) 96‐, and (v) 120‐h post‐inoculation of H129‐EGFP+ into the colorectal wall. Scale bars: 100 μm. (vi) Quantification of the number of H‐129 EGFP+ cells within sections of DRG. **p < 0.01 and ****p < 0.0001 determined by a one‐way Kruskal‐Wallis test (non‐parametric data), Dunn's multiple comparisons tests were used to compare between time points. Data points represent the individual DRG sections, gained from 2 to 10 sections/mouse, collected 24 h (*N* = 3 mice, 2F:1M), 48 h (*N* = 4 mice, 2F:2M), 72 h (*N* = 4 mice, 2F:2M), 96 h (*N* = 3 mice, 1F:2M), and 120 h (*N* = 3, 2F:1M) post‐HSV‐1 H129‐EGFP inoculation. Error bars represent the standard error of the mean.

**FIGURE 3:**
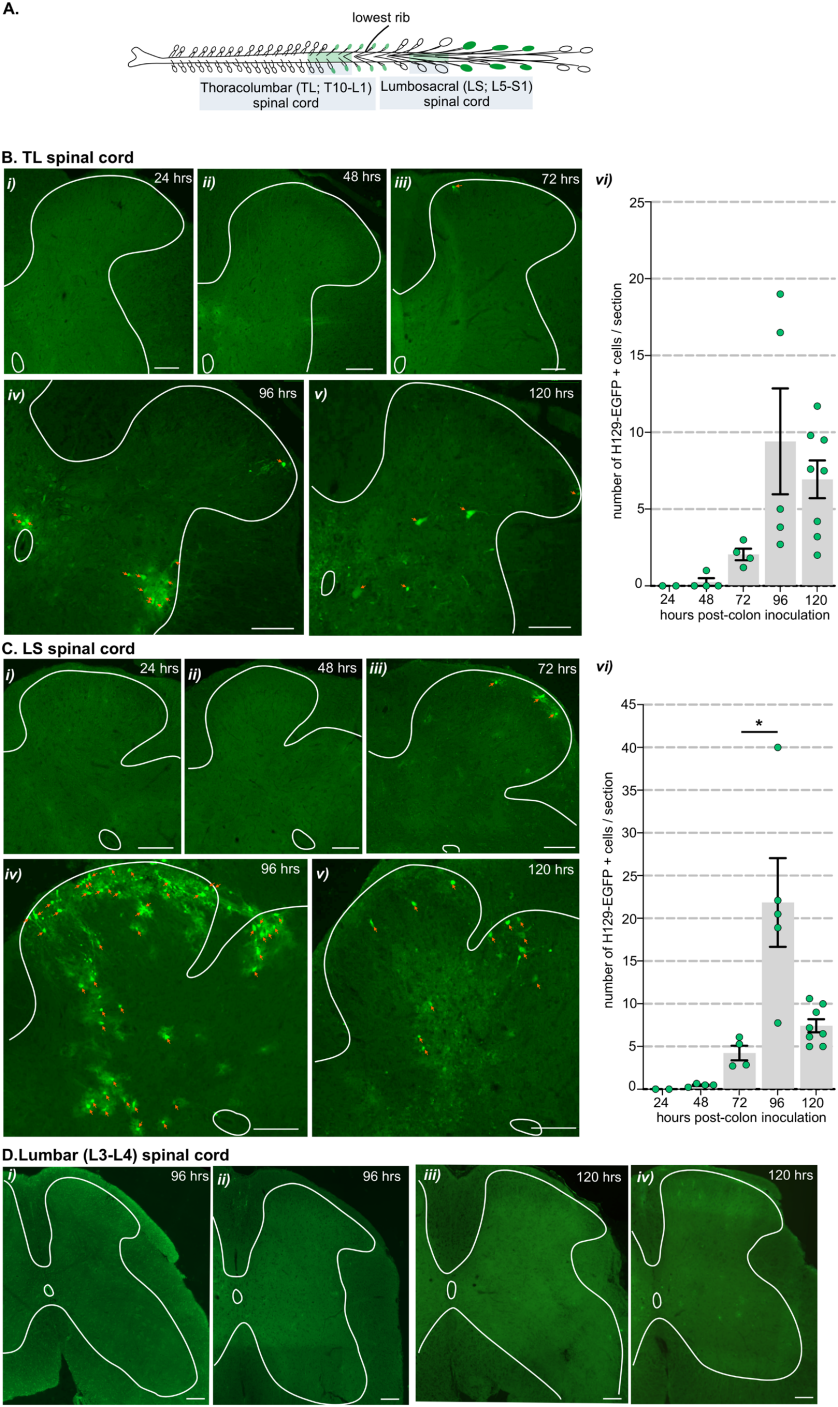
Distribution of H129‐EGFP+ cells within the thoracolumbar (TL) and lumbosacral (LS) spinal cord dorsal horn following colorectal HSV‐1 H129‐EGFP inoculation. (A) Schematic showing the spinal cord levels known to receive input from colorectal sensory afferent neurons, thoracic 10 through lumbar 1 (thoracolumbar, TL, T10‐L1) and lumbar 5 through sacral 1 (lumbosacral, LS. L5‐S1), in which HSV‐1 H129‐EGFP‐labeling was assessed in 24‐h increments after colorectal inoculation. (B) Representative photomicrographs of H129‐EGFP+ cells (green, indicated by orange arrows) within sections of TL spinal cord dorsal horn (i) 24‐, (ii) 48‐, (iii) 72‐, (iv) 96‐, and (v) 120‐h post‐inoculation of H129‐EGFP+ within the colorectal wall. Scale bars: 100 μm. (vi) Quantification of the mean number of H‐129 EGFP+ cells within sections of TL spinal cord dorsal horn. One‐way ANOVA (parametric data) with Bonferroni multiple comparison tests was used to compare between time points. Data points represent the mean number of H129‐EGFP+ cells/section of individual mice, gained from 5 to 10 sections/mouse, collected 24 h (*N* = 2 mice, 2F), 48 h (*N* = 4 mice, 2F:2M), 72 h (*N* = 4 mice, 2F:2M), 96 h (*N* = 5 mice, 3F:2M), and 120 h (*N* = 8, 7F:1M) post‐HSV‐1 H129‐EGFP inoculation. Error bars represent the standard error of the mean. (C) Representative photomicrographs of H129‐EGFP+ cells (green, indicated by orange arrows) within sections of LS spinal cord dorsal horn (i) 24‐, (ii) 48‐, (iii) 72‐, (iv) 96‐, and (v) 120‐h post‐inoculation of H129‐EGFP+ within the colorectal wall. Scale bars: 100 μm. (vi) Quantification of the mean number of H‐129 EGFP+ cells within sections of the LS spinal cord dorsal horn. *p < 0.05, determined by one‐way ANOVA (parametric data) with Bonferroni multiple comparison tests. Data points represent the mean number of H129‐EGFP+ cells/section of individual mice, gained from 5 to 10 sections/mouse, collected 24 h (*N* = 2 mice, 2F), 48 h (*N* = 4 mice, 2F:2M), 72 h (*N* = 4 mice, 2F:2M), 96 h (*N* = 5 mice 3F:2M) and 120 h (*N* = 8, 7F:1M) post‐HSV‐1 H129‐EGFP inoculation. Error bars represent the standard error of the mean. (D) Representative photomicrographs of lumbar spinal cord (L3‐L4) (i, ii) 96‐ and (iii–iv) 120‐h post‐inoculation of H129‐EGFP+ within the colorectal wall. Scale bars: 100 μm.

**FIGURE 4:**
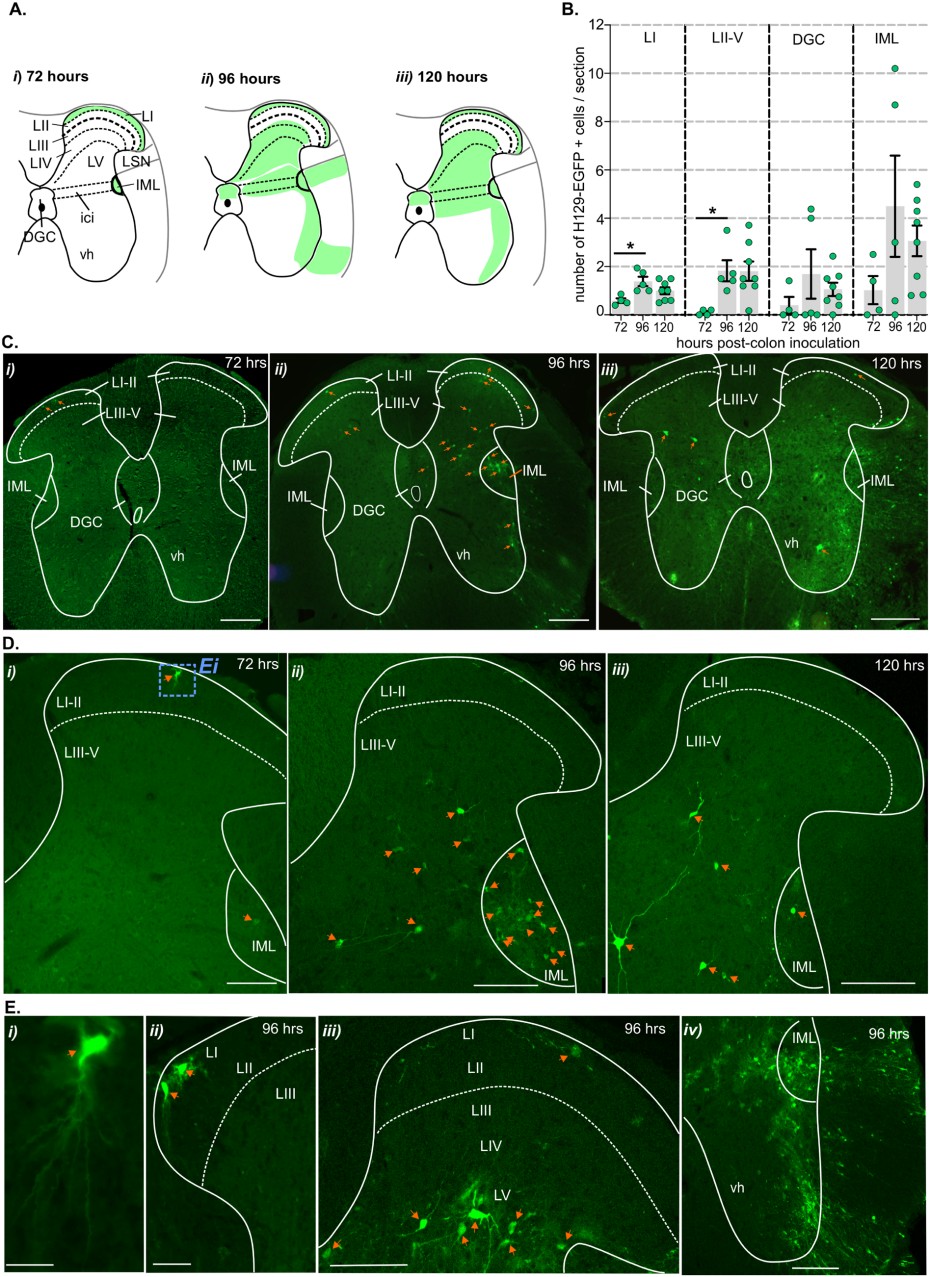
Distribution of H129‐EGFP+ cells within the thoracolumbar (TL) spinal cord dorsal horn following colorectal HSV‐1 H129‐EGFP inoculation. (A) Schematic (Lein et al. 2007) illustration of the TL spinal cord summarizing the distribution (regions indicated by green shading) of H129‐EGFP+ cells 72‐, 96‐, and 120 h following HSV‐1 H129‐EGFP inoculation of the colorectum. (B) Quantification of the mean number (#) of H‐129 EGFP+ cells within sections of TL spinal cord dorsal horn in lamina I (LI), laminae II–V (LII–V), dorsal gray commissure (DGC), and the intermediolateral nuclei (IML) compared between tissue collected 72‐, 96‐, 120 h post‐HSV‐1 H129‐EGFP colorectal inoculation. *p < 0.05, determined by one‐way ANOVA (parametric data) with Bonferroni multiple comparison tests. Data points represent the mean number of H129‐EGFP+ cells/section of individual mice, gained from 5 to 10 sections/mouse collected 72 h (*N* = 4 mice, 2F:2M), 96 h (*N* = 5 mice 3F:2M) and 120 h (*N* = 8 mice, 7F:1M) post‐HSV‐1 H129‐EGFP inoculation. Error bars represent the standard error of the mean. (C, D) Representative photomicrographs of the TL spinal cord dorsal horn at (C) low magnification and (D) high magnification showing the distribution of H129‐EGFP+ cells (green, highlighted by orange arrows) in tissue collected at (i) 72‐, (ii) 96‐, and (iii) 120 h after colorectal inoculation. Scale bars: 100 μm. (E) Photomicrographs showing the distribution of EGFP‐labeled cells (green, highlighted by orange arrows) within the (i and ii) superficial dorsal horn laminae I–II (LI–II), (iii) dorsal horn laminae I–V (LI–V), and (iv) ventral horn in tissue collected at (i) 72‐ and (ii–iv) 96‐h post‐inoculation. The image in Ei (scale bar: 20 μm) corresponds to the region within the blue dotted box in Di. Scale bars: 100 μm. Dashed lines indicate the border between lamina II (LII) and lamina III (LIII). Abbreviations: ici, intercalated nuclei; LI, lamina 1; LII, lamina 2; LIII, lamina 3; LIV, lamina 4; LV, lamina 5; IML, intermediolateral nuclei and vh, ventral horn.

**FIGURE 5:**
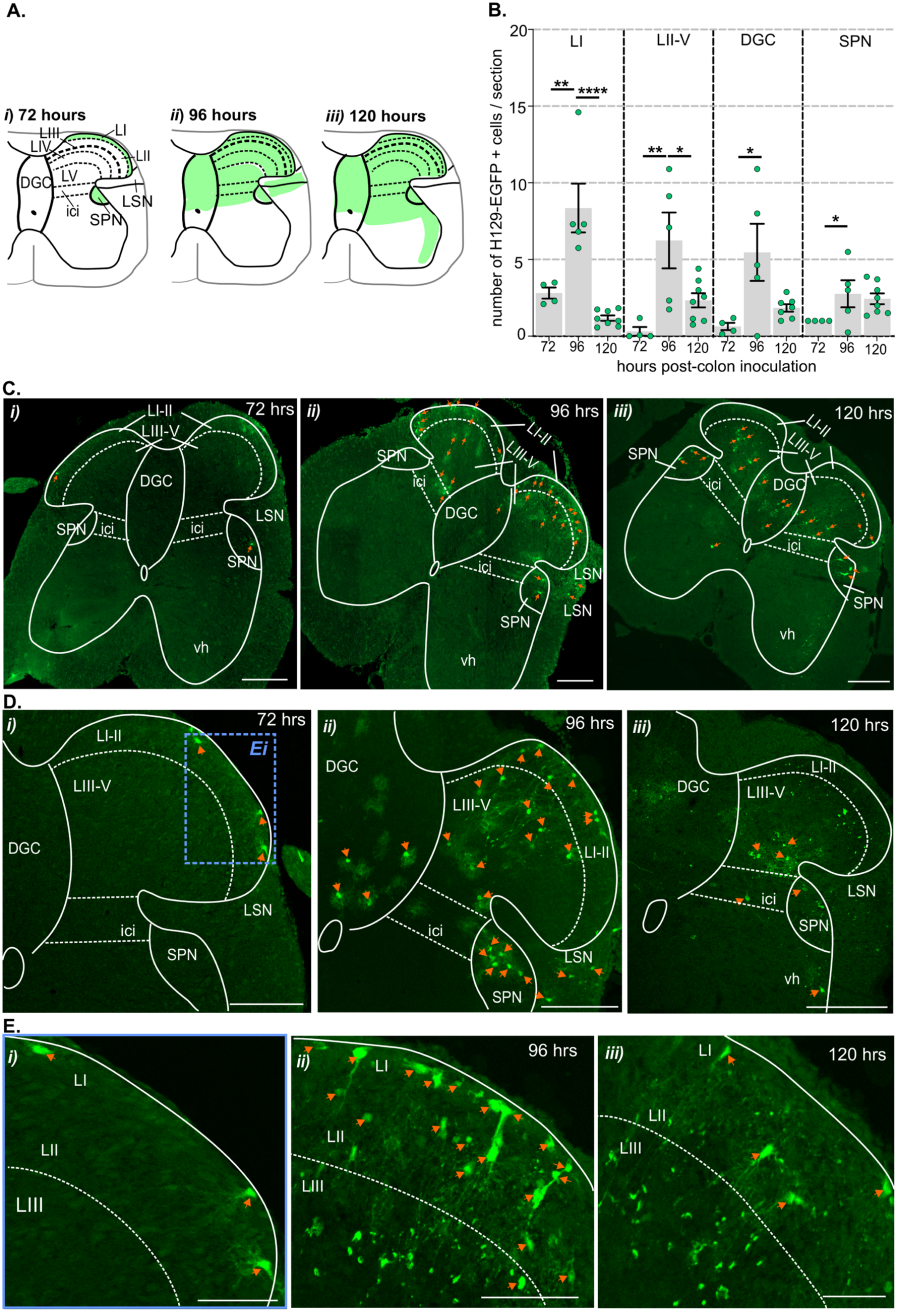
Distribution of H129‐EGFP+ cells within the lumbosacral (LS) spinal cord dorsal horn following colorectal HSV‐1 H129‐EGFP inoculation. (A) Schematic (Lein et al. 2007) illustration of the LS spinal cord summarizing the distribution (regions indicated by green shading) of H129‐EGFP+ cells at 72‐, 96‐, and 120 h following HSV‐1 H129‐EGFP colorectal inoculation. (B) Quantification of the mean number of H‐129 EGFP+ cells within sections of LS spinal cord dorsal horn in lamina I (LI), laminae II–V (LII–V), dorsal gray commissure (DGC), and the sacral parasympathetic nucleus (SPN) compared between tissue collected 72‐, 96‐, and 120‐h post‐HSV‐1 H129‐EGFP inoculation. *p < 0.05, **p < 0.01, and ****p < 0.0001, determined by one‐Way ANOVA (parametric data) with Bonferroni multiple comparison tests. Data points represent the mean number of H129‐EGFP+ cells/section of individual mice, gained from 5 to 10 sections/mouse collected 72 h (*N* = 4 mice, 2F:2M), 96 h (*N* = 5 mice, 3F:2M) and 120 h (*N* = 8 mice, 7F:1M) post‐ HSV‐1 H129‐EGFP inoculation. Error bars represent the standard error of the mean. (C, D) Representative photomicrographs of the LS spinal cord dorsal horn at (C) low magnification and (D) high magnification showing the distribution of H129‐EGFP+ cells (green, highlighted by orange arrows) in tissue collected at (i) 72‐, (ii) 96‐, and (iii) 120 h after colorectal inoculation. Scale bars: 100 μm. (E) Photomicrographs of the LS spinal cord at high magnification showing the distribution of H129‐EGFP+ cells (green, highlighted by orange arrow) within the superficial dorsal horn laminae I–III (LI–III), in tissue collected at (i) 72‐, (ii) 96‐, and (iii) 120 h post‐inoculation of HSV‐1 H129‐EGFP within the colorectal wall. Image (Ei) corresponds to the region within the blue dotted box in (Di). Scale bars: 20 μm. Dashed lines indicate the border between lamina II (LII) and lamina III (LIII) and the lateral edges of the intercalated nuclei (ici). Abbreviations: DGC, dorsal gray commissure; LI, lamina 1; LII, lamina 2; LIII, lamina 3; LIV, lamina 4; LV, lamina 5; LSN, lateral spinal nucleus; SPN, sacral parasympathetic nuclei, and vh, ventral horn.

**FIGURE 6:**
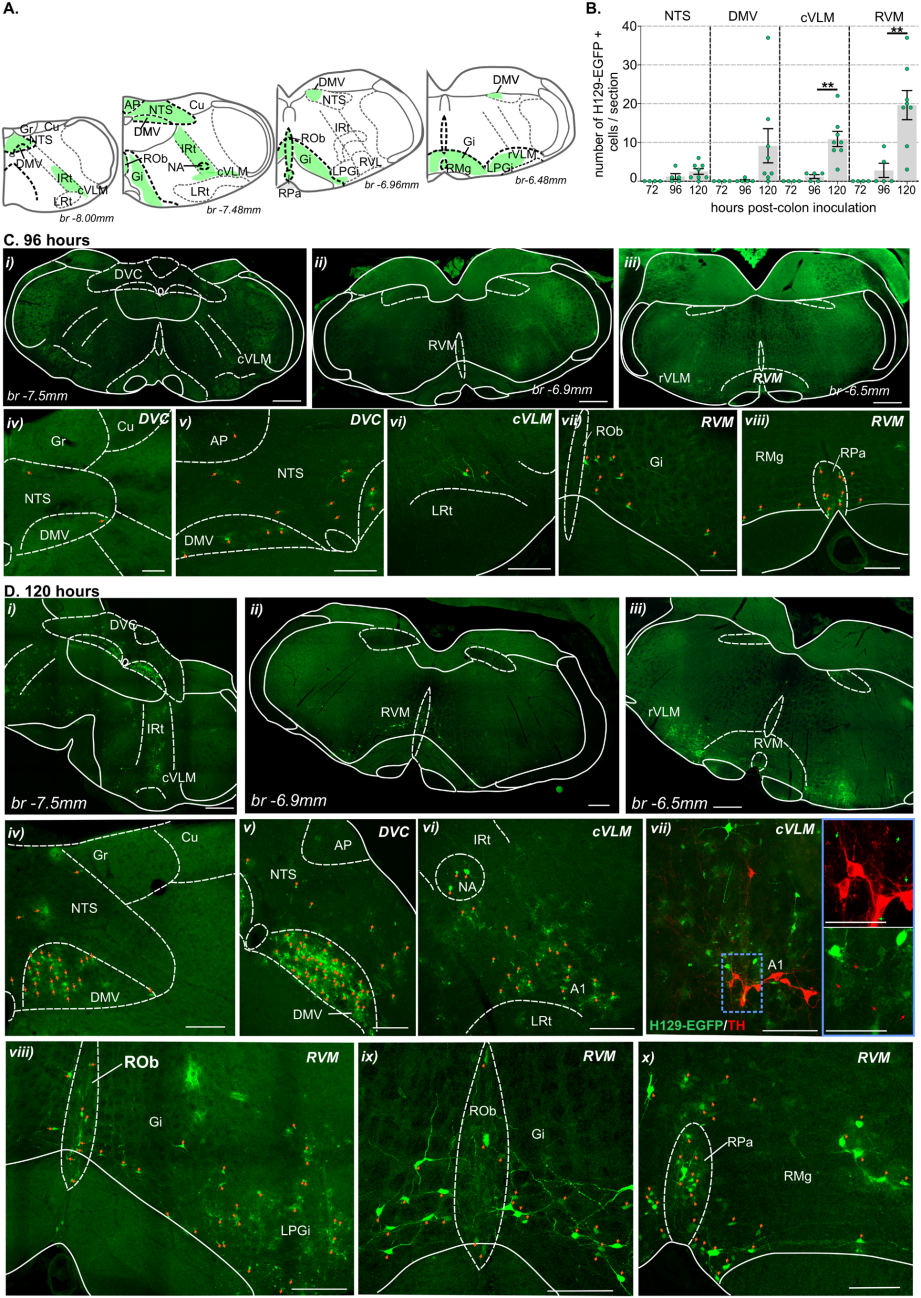
Distribution of H129‐EGFP+ cells in the medulla post‐colorectal HSV‐1 H129‐EGFP inoculation. (A) Schematic representation of the medulla (running caudal to rostral in the left‐to‐right axis) (Paxinos and Franklin 2001) summarizing distribution (regions indicated by green shading) of H129‐EGFP+ cells 120 h following HSV‐1 H129‐EGFP colorectal inoculation. (B) Quantification of the mean number of H‐129 EGFP+ cells within sections of the nucleus of the solitary tract (NTS), dorsal motor nucleus of the vagus (DMV), caudal ventrolateral medulla (cVLM), and rostral ventromedial medulla (RVM) compared between tissue collected at 72‐, 96‐, and 120 h post‐HSV‐1 H129‐EGFP colorectal inoculation. **p < 0.01, determined by one‐Way ANOVA (parametric data) with Bonferroni multiple comparison tests. Data points represent the mean number of H129‐EGFP+ cells/section of individual mice, gained from 2 to 3 sections/mouse collected 72 h (*N* = 4 mice, 2F:2M), 96 h (*N* = 5 mice, 3F:2M), and 120 h (*N* = 8 mice, 7F:1M) post‐inoculation. Error bars represent the standard error of the mean. (C) Photomicrographs at (i–iii) low and (iv–viii) high magnification of (i) caudal, (ii) intermediate, and (iii) rostral sections of the medulla showing the distribution of H129‐EGFP+ cells (green, highlighted by orange arrows) in the (i, iv, v) dorsal vagal complex (DVC) and within the reticulum of the (i, vi) cVLM and (ii, iii, vii, viii) RVM 96 h post‐inoculation of HSV‐1 H129‐EGFP within the colorectal wall. Scale bars: i–iii: 500 μm and iv–vi: 100 μm. (D) Photomicrographs at (i–iii) low and (iv–x) high magnification of (i, iv–vii) caudal, (ii, viii, ix) intermediate, and (iii, x) rostral sections of the medulla showing the distribution of H129‐EGFP+ cells (green, highlighted by orange arrows) in the (i, iv, v) DVC, (i, vi, vii) cVLM and (ii, iii, viii, ix, x) rostral ventrolateral medulla (rVLM) and RVM 120‐h post‐inoculation of the colorectal wall with HSV‐1 H129‐EGFP. (vii) Photomicrograph of the cVLM immunolabeled for tyrosine hydroxylase (TH, red); EGFP‐labeled cells are indicated by green arrows, and TH‐immunoreactive neurons are indicated by red arrows. Images in the blue‐lined box (scale bar: 50 μm) correspond to the region within the blue dotted box. Scale bars: i–iii: 500 μm and iv–x: 150 μm. Abbreviations: AP, Area postrema; br, bregma; Cu, cuneate nuclei; DMV, dorsal motor nucleus of the vagus; Gi, gigantocellular reticular nucleus; Gr, gracile nuclei; IRt, intermediate reticular nucleus; LPGi, lateral paragigantocellular nucleus; LRt, lateral reticular nucleus; NA, nucleus ambiguus; NTS, nucleus of the solitary tract; RMg, raphe magnus nucleus; ROb, raphe obscurus nucleus; RPa, raphe pallidus nucleus; rVLM, rostroventrolateral reticular nucleus.

**FIGURE 7:**
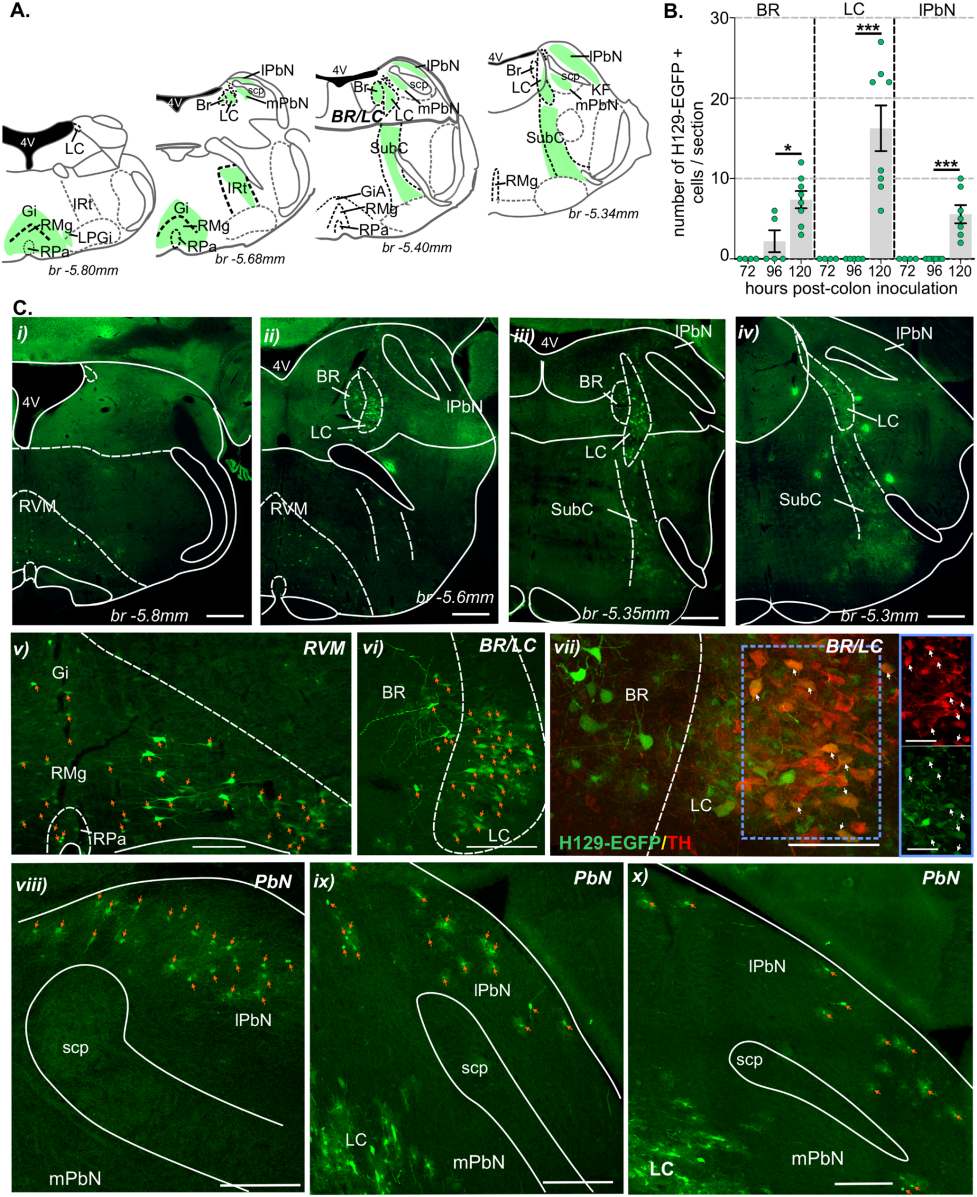
Distribution of H129‐EGFP+ cells in the pons post‐colorectal HSV‐1 H129‐EGFP inoculation. (A) Schematic representation of the pons (running caudal to rostral in left‐to‐right axis) (Paxinos and Franklin 2001) summarizing distribution (regions with indicated by green shading) of H129‐EGFP+ cells 120 h following HSV‐1 H129‐EGFP colon inoculation. (B) Quantification of the mean number of H‐129 EGFP+ cells within sections of Barrington's nucleus (BR), locus coeruleus (LC), and lateral parabrachial nucleus (lPbN) compared between tissue collected 72‐, 96‐, 120 h post‐HSV‐1 H129‐EGFP colorectal inoculation. *p < 0.05, ***p < 0.001, determined by one‐way ANOVA (parametric data) with Bonferroni multiple comparison tests. Data points represent the mean number of H129‐EGFP+ cells/section of individual mice, gained from 2 to 5 sections/mouse collected 72 h (*N* = 4 mice, 2F:2M), 96 h (*N* = 5 mice, 3F:2M), and 120 h (*N* = 8 mice, 7F:1M) post‐inoculation. Error bars represent the standard error of the mean. (C) Photomicrographs at (i–iv) low and (v–x) high magnification of pontine sections from mice at 120 h post‐inoculation of HSV‐1 H129‐EGFP within the colorectal wall. Images demonstrate the distribution of H129‐EGFP+ cells (green, highlighted by orange arrows) within the (i, ii, v) rostral ventromedial medulla (RVM), (ii–iv, vi, vii) BR, LC, and subcoeruleus (subC), and the (ii–iv, viii–x) lPbN. (vii) Photomicrograph of the BR and LC immunolabeled for tyrosine hydroxylase (TH, red); EGFP/TH co‐labeled neurons are highlighted by white arrows. The image in the blue‐lined box (scale bar: 20 μm) corresponds to the region within the blue‐dotted box. Scale bars: i–iv: 500 μm, v, vi, viii–x: 150 μm, and vii: 50 μm. Abbreviations: 4 V, Fourth ventricle; br, bregma; Gi, gigantocellular reticular nucleus; IRt, intermediate reticular nucleus; KF, Kölliker‐Fuse nucleus; lPbN, lateral parabrachial nuclei; LPgi, lateral paragigantocellular nucleus; mPbN, medial parabrachial nuclei; RMg, raphe magnus nucleus; RPa, raphe pallidus nucleus; scp, superior cerebellar peduncle.

**FIGURE 8:**
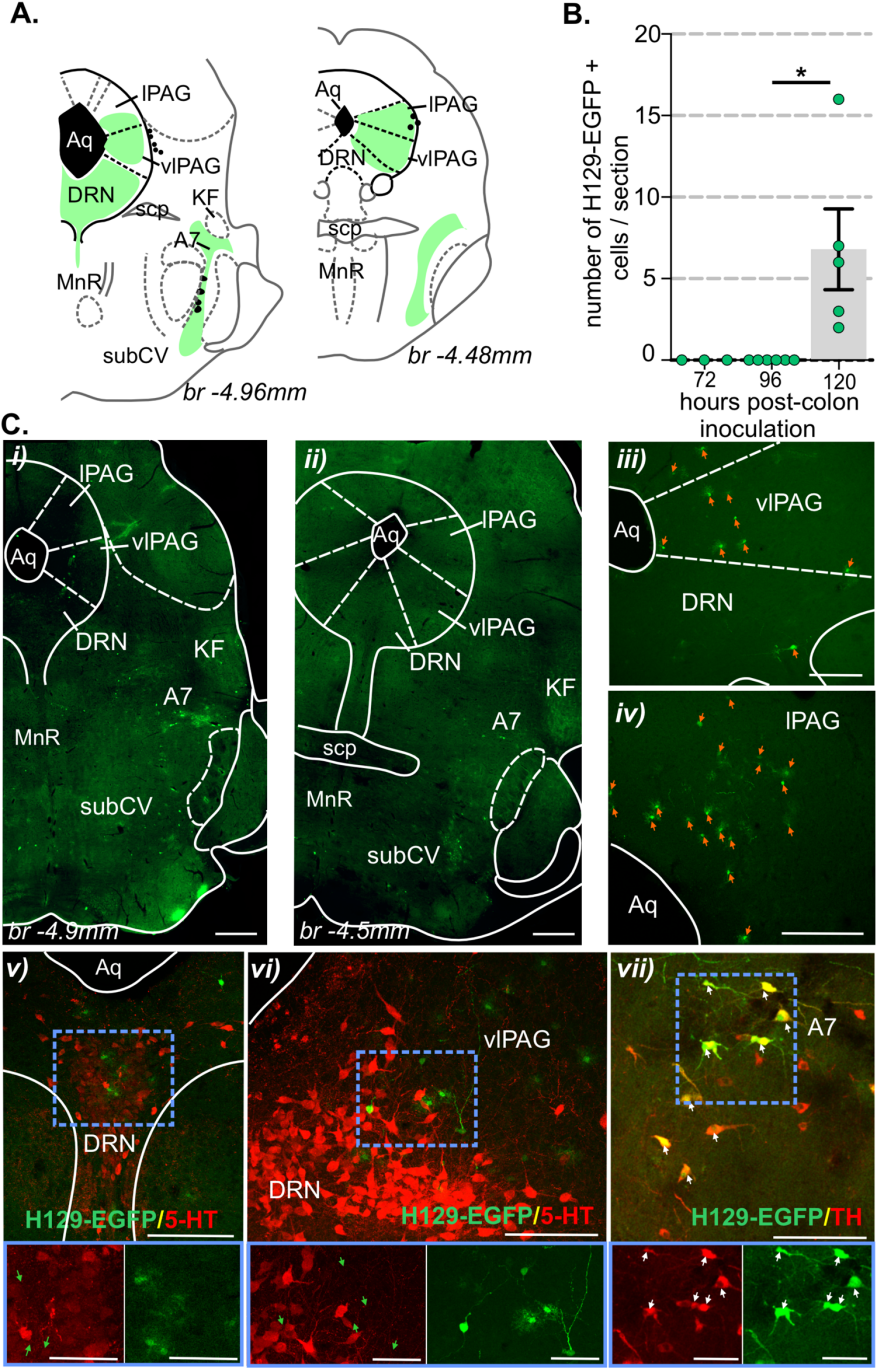
Distribution of H129‐EGFP+ cells in the midbrain post‐colorectal HSV‐1 H129‐EGFP inoculation. (A) Schematic representation of the caudal midbrain (running caudal to rostral in left to right axis) (Paxinos and Franklin 2001) summarizing distribution (regions indicated by green shading) of H129‐EGFP+ cells 120 h following HSV‐1 H129‐EGFP colon inoculation. (B) Quantification of the mean number of H‐129 EGFP+ cells within sections of the ventrolateral (vlPAG) and lateral (lPAG) periaqueductal gray compared between tissue collected 72‐, 96‐, and 120 h post‐HSV‐1 H129‐EGFP colorectal inoculation. *p < 0.05, determined by one‐way ANOVA (parametric data) with Bonferroni multiple comparison tests. Data points represent the mean number of H129‐EGFP+ cells/section of individual mice, gained from 2 to 5 sections/mouse collected 72 h (*N* = 4 mice, 2F:2M), 96 h (*N* = 5 mice, 3F:2M), and 120 h (*N* = 5 mice, 5F) post‐inoculation. Error bars represent the standard error of the mean. (C) Photomicrographs at (i, ii) low and (iii, vii) high magnification of midbrain sections from mice at 120 h post‐inoculation of HSV‐1 H129‐EGFP within the colorectal wall, demonstrating the distribution of H129‐EGFP+ cells (green, highlighted by orange arrows) within the (i–vi) periaqueductal gray (PAG) and the dorsal raphe nuclei (DRN), and the (i, ii, vii) A7. (v, vi) Photomicrographs of the PAG and DRN immunolabeled for serotonin (5‐HT, red). H129‐EGFP+ cells are indicated by green arrows. The image in the blue‐lined box (scale bar: 50 μm) corresponds to the region within the blue‐dotted box. (vii) Photomicrograph of the A7 immunolabeled for tyrosine hydroxylase (TH, red), EGFP/TH co‐labeled neurons are highlighted by white arrows. Images in the blue‐lined box (scale bar: 50 mm) correspond to the region within the blue dotted box. Scale bars: i, ii: 500 μm and iii, vii: 100 μm. Abbreviations: Aq, aqueduct; Br, bregma; KF, Kölliker‐Fuse nucleus; lPAG, lateral periaqueductal gray; MnR, median raphe nucleus; scp, superior cerebellar peduncle; SubCV, subcoeruleus ventral; vlPAG, ventrolateral periaqueductal gray.

Following localized injections of HSV‐1 H129‐EGFP into the colorectal wall (Figure S1), we tracked, in 24‐h increments, when H129‐EGFP+ cells appeared in the DRG and spinal cord at levels associated with the spinal afferent innervation of the colorectum, relative to when H129‐EGFP+ cells appeared in the brainstem (Figure 1). From 48 h post‐inoculation, H129‐EGFP+ cells were observed in DRG (Figure 1 and Figure 2) at TL (T10‐LI; Figure 1 and Figure 2A,B) and LS (L6‐S1; Figure 1 and Figure 2A,C) spinal levels. The number of H129‐EGFP+ cells was greatest in the LS DRG and the density varied over time post‐inoculation (Figure 1 and Figure 2Bvi,Cvi). H129‐EGFP+ cells were observed in the relevant spinal cord segments (Figure 3A) from 72 h post‐inoculation (Figure 1 and Figures 3, 4, 5). Very few, if any, H129‐EGFP+ cells were observed in the spinal cord collected 24‐ and 48‐h post‐inoculation (Figure 1 and Figure 3Bi,Bii,Bvi,Ci,Cii,Cvi). H129‐EGFP+ cells were not observed in the dorsal horn at lumbar spinal cord segments L3‐L4, which lack input from the colorectum (Figure 3D). These findings align with our previous study showing DRG at spinal levels T10‐L1 and L5‐S1 contain colorectal afferent neurons, at greater density in the LS DRG, and that DRG at L3‐4 do not contain colonic afferent neurons (Wang et al. 2024). In the spinal cord, H129‐EGFP+ cells were predominantly localized to the dorsal horn, the density and distribution of which varied between the TL (Figure 1, Figure 3B, and Figure 4) and LS (Figure 1, Figure 3C, and Figure 5) spinal segments and over time post‐inoculation. In the brainstem, H129‐EGFP+ cells were observed in discrete nuclei within the medulla (Figure 1 and Figure 6) 96‐ and 120 h post‐inoculation and in the pons (Figure 1 and Figure 7) and caudal midbrain (Figure 1 and Figure 8) 120 h post‐inoculation. In the spinal cord and brainstem, H129‐EGFP+ cells had varied morphologies, suggesting labeling of neuronal cell bodies and their processes and glial cells as previously reported (Garcia‐Luna et al. 2021).

In the TL spinal cord dorsal horn 72 h post‐inoculation, H129‐EGFP+ cells were distributed in the superficial dorsal horn lamina I (LI, Figure 3Biii and Figure 4Ai,B,Ci,Di,Ei) and the intermediolateral nuclei (IML, Figure 4Ai,B,Di). In tissue collected 96–120 h post‐inoculation, the number of H129‐EGFP+ cells in LI and IML increased relative to 72 h (Figure 4B) and were additionally localized in deeper dorsal horn laminae III–V and dorsal to the central canal in the DGC (or LX) (Figure 3Biv,Bv, and Figure 4Aii,Aiii,B,Cii,Ciii,Dii,Diii,Eii,Eiii). H129‐EGFP+ labelling was also observed in the ventrolateral edges of the ventral horn and within white matter tracts (Figure 4Aii,Aiii,Cii,Ciii,Eiv). In the LS spinal cord (Figure 3C and Figure 5), 72 h post‐inoculation H129‐EGFP+ cells were evident in the dorsal horn LI (Figure 3 Ciii and Figure 5Ai,B,Ci,Di,Ei) and the SPN (Figure 5Ai,B,Ci). In tissue collected 96 h pos,t‐inoculation the number of H129‐EGFP+ cells significantly increased, relative to 72 h, throughout LI–V (Figure 3Civ and Figure 5Aii,B,Cii,Dii,Eii) and within the DGC and the SPN (Figure 3Civ and Figure 5Aii,B,Cii,Dii). 120 h post‐inoculation, H129‐EGFP+ labelling in the LS dorsal horn was fragmented (Figure 3Cv and Figure 5Ciii,Diii,Eiii) and fewer H129‐EGFP+ cells were evident in the dorsal horn LI–V and the DGC relative to 96 h post‐inoculation (Figure 5B). The numbers of H129‐EGFP+ cells in the SPN was similar 96‐ and 120 h post‐inoculation (Figure 5B) and labelling was also evident in ventrolateral tracts of the ventral horn 120 h post‐inoculation (Figure 5Ciii,Diii).

H129‐EGFP+ cells were evident in the medulla 96 h post‐inoculation, and their density and distribution spread 120 h post‐inoculation within specific nuclei and reticular formations (Figure 6A,B). 96 h post‐inoculation H129‐EGFP+ cells (Figure 6C) were observed in the nucleus of the solitary tract (NTS) and dorsal motor nucleus of the vagus (DMV) of the caudal medulla dorsal vagal complex (DVC, Figure 6B,Ci,Civ,Cv and Figure S2). 120 h post‐inoculation (Figure 6D) the density of H129‐EGFP+ cells within the NTS was unchanged from that observed 96 h afterinoculation, but increased within the DMV (Figure 6B,Di,Div,Dv and Figure S2). H129‐EGFP+ cells were also localized within the reticulum of the caudal (cVLM) and rostral ventrolateral medulla (rVLM) and the rostral ventromedial medulla (RVM) (Figure 6A). Within the cVLM, H129‐EGFP+ cells were evident in the lateral reticulum in the area of the nucleus ambiguus (NA) and the intermediate reticular nucleus (IRt) (Figure 6Ci,Cvi,Di,Dvi and Figure S3A). As well as in reticular formation dorsal to the lateral reticular nucleus (LRt, Figure 6Ci,Cvi,Di,Dvi) surrounding catecholamine (tyrosine hydroxylase (TH)‐immunoreactive (IR)) A1 cell group, but not TH‐IR themselves (Figure 6Dvii). In the rostral medulla, H129‐EGFP+ cells were evident in the reticulum of the ventrolateral medulla (Figure 6Cii,Ciii,Dii,Diii) and the RVM raphe obscurus nucleus (ROb), gigantocellular reticular nucleus (Gi), lateral paragigantocellular nucleus (LPGi, Figure 6Cii,Civ,Dii,Dviii,Dix, and Supplementary Figure 3B), the raphe magnus nucleus (RMg), and the raphe pallidus nucleus (RPa, Figure 6Ciii,Cviii,Diii,Dx, and Figure S3B).

H129‐EGFP+ cells were observed in distinct structures within the pons 120 h post‐inoculation (Figure 7A,B and Figure S3C,D). Specifically, within the RVM reticulum (Figure 7A,Ci,Cii,Cv), Barrington's nucleus (BR) and the locus coeruleus (LC, Figures 7A,B,Cii,Ciii,Cvi,Cvii, and Figure S3C), the subcoeruleus (SubC, Figure 7A,Ciii,Civ) and the dorsal aspect of the lateral parabrachial nuclei (lPbn, Figure 7A,B,Cii–iv,Cviii–x and Figure S3D). A few H129‐EGFP + cells were observed in Barrington's nucleus (BR) 96 h post‐inoculation, the number of which significantly increased 120 h post‐inoculation (Figure 7B and Figure S3C). H129‐EGFP+ cells were observed in the caudal midbrain 120 h post‐inoculation (Figure 8 and Figure S3E). Specifically, within the lateral (lPAG) and ventrolateral (vlPAG) sub‐regions of the periaqueductal gray (PAG, Figure 8A,B,Ci–iv,Cvi). H129‐EGFP + cells were also evident in the dorsal raphe nuclei (DRN, Figure 8A,Ci–iii,Cv), the ventral aspect of the SubC (Figure 8A,Ci,Cii) and within the A7 catecholamine cellular region, where they co‐labeled for TH (Figure 8A,Ci,Cii,Cvii).

### 3.2 Distribution of pERK‐Immunoreactive (IR) Neurons in the Brainstem Following Noxious Colorectal Distension (CRD)

**FIGURE 9:**
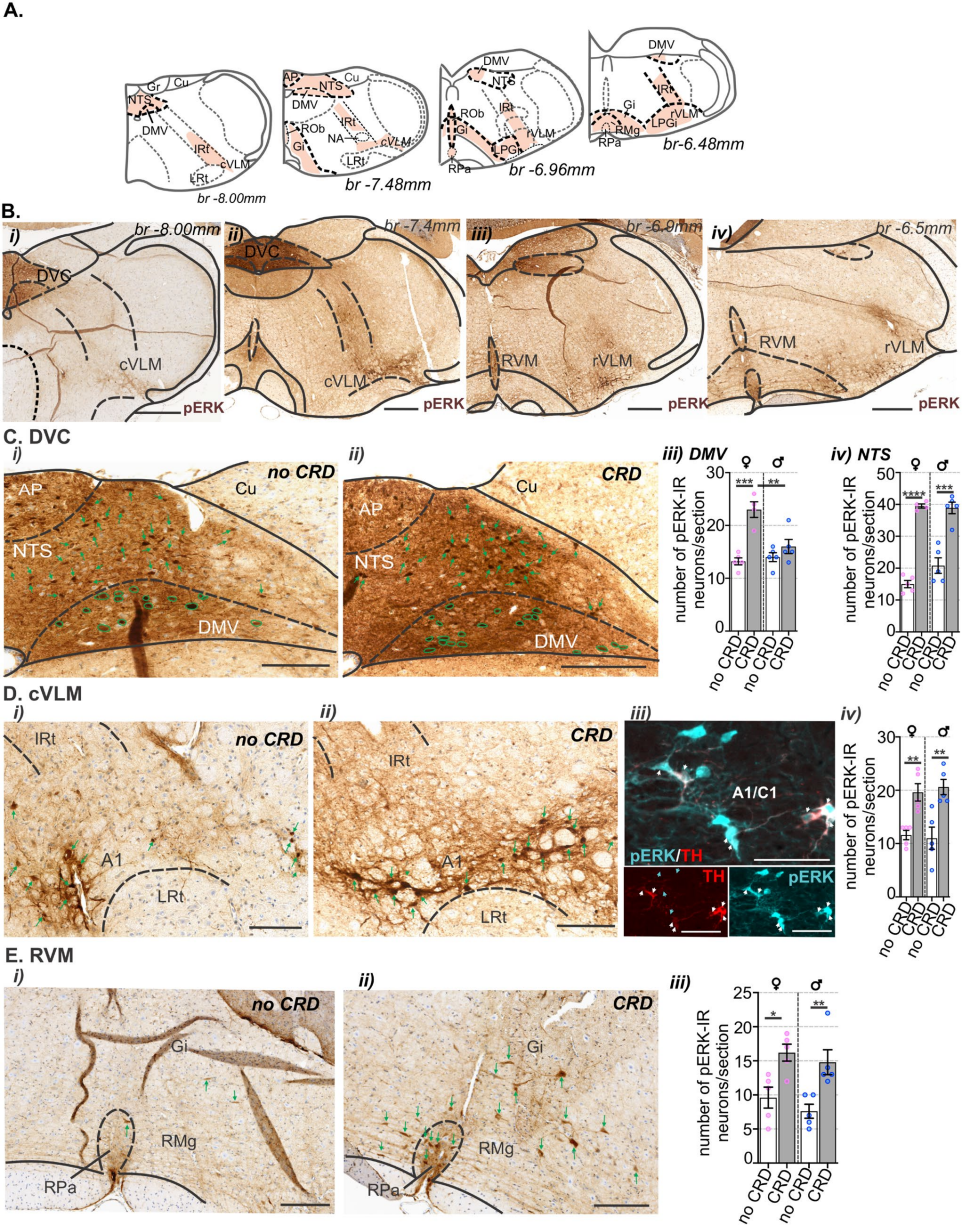
Distribution of pERK‐immunoreactive (pERK‐IR) neurons in the medulla following in vivo colorectal distension (CRD). (A) Schematic representation of the medulla oblongata (Paxinos and Franklin 2001) summarizing the distribution (regions indicated by orange shading) of pERK‐IR neurons following in vivo noxious CRD. (B) Low magnification photomicrographs of (i, ii) caudal, (iii) intermediate, and (iv) rostral sections of the medulla showing the distribution of pERK‐IR neurons (brown) following in vivo CRD at noxious pressures. Scale bars: 500 μm. (C–E) High magnification photomicrographs showing the distribution of pERK‐IR neurons (brown cells, indicated by green outline or green arrows) within the (C) caudal dorsal vagal complex (DVC), (D) caudal ventrolateral medulla (cVLM), and (E) rostral ventromedial medulla (RVM) from mice that underwent (i) no CRD or (ii) noxious CRD (CRD). (Diii) Photomicrograph of the A1 nuclei within the cVLM immunolabeled for pERK (cyan) and tyrosine hydroxylase (TH, red); pERK‐immunoreactive neurons are indicated by cyan arrows, and pERK/TH co‐labeled cells are indicated by white arrows. Scale bars: = 100 μm. Quantification comparing the number of pERK‐IR neurons between female (♀, pink data points) and male (♂, blue data points) mice that underwent no CRD (white bar) or noxious CRD (gray bar) in sections of (C) DVC, (Ciii) dorsal vagal motor nuclei (DMV) and (Civ) nucleus of the solitary tract (NTS), (Diii) cVLM (inclusive of the A1 region ventral to the intermediate reticular (IRt), and dorsal to the lateral reticular nucleus (LRt)) and (Eiii) RVM (inclusive of the raphe pallidus nucleus (RPa), raphe magnus nucleus (RMg), and the gigantocellular reticular nucleus (Gi)). *p < 0.05, **p < 0.01, ***p < 0.001, ****p < 0.0001, determined by unpaired t‐tests. Data points represent the mean number of pERK‐IR neurons/section of individual mice, gained from 1 to 55 sections/mouse from No CRD: *N* = 5F:5M mice and CRD: NTS and DMV 4F:5M mice; cVLM and RVM 5F:5M mice. Error bars represent the standard error of the mean. Abbreviations: AP, Area postrema; br, bregma; Cu, cuneate nuclei; Gr, gracile nuclei; LPGi, lateral paragigantocellular nucleus; LRt, lateral reticular nucleus; NA, nucleus ambiguus; ROb, raphe obscurus nucleus; rRVM, rostroventrolateral reticular nucleus.

While care was taken to avoid leakage of the viral tracer, it is a possibility that some off‐target sites were exposed to the virus and resulted in labeling in the brainstem not relevant to the colorectum. To assess this, the distribution of neurons in the brainstem activated by in vivo CRD, identified by pERK‐immunoreactivity relative to no CRD controls, was used to identify brainstem nuclei functionally relevant to colorectal processing and to compare to the distribution of H129‐EGFP+ cells. Following noxious CRD, similar to the distribution of H129‐EGFP+ cells, pERK‐IR neurons were observed within specific nuclei of the caudal medulla, in the NTS and DMV and in the cVLM (Figure 9A,Bi,Bii,D). In contrast to the density of H129‐EGFP+ cells, pERK‐IR neurons were relatively dense within the NTS (Figure 9Bi,Bii,C) and populations of pERK‐IR neurons within the cVLM were immunolabeled for TH (Figure 9Diii). In the NTS (Figure 9C) and the cVLM (Figure 9D), compared to no CRD controls (Figure 9Ci,Di), noxious CRD (Figure 9Cii,Dii) evoked significant increases in the number of pERK‐IR neurons in both male and female mice (Figure 9Civ,Div). A significant difference between female and male mice was observed in the DMV, with a greater number of pERK‐IR neurons evident in female mice following noxious CRD than male mice (Figure 9Ciii). In the rostral medulla (Figure 9A,Biii,Biv), pERK‐IR neurons were similarly distributed as H129‐EGFP+ cells within the reticular of the rVLM (Figure 9A,Biii,Biv) and RVM (Figure 9A,Biii,Biv,E). In the RVM (Figure 9E), compared to no CRD controls (Figure 9Ei), noxious CRD (Figure 9Eii) evoked a significant increase in the number of pERK‐IR neurons in both male and female mice (Figure 9Eiii).

**FIGURE 10:**
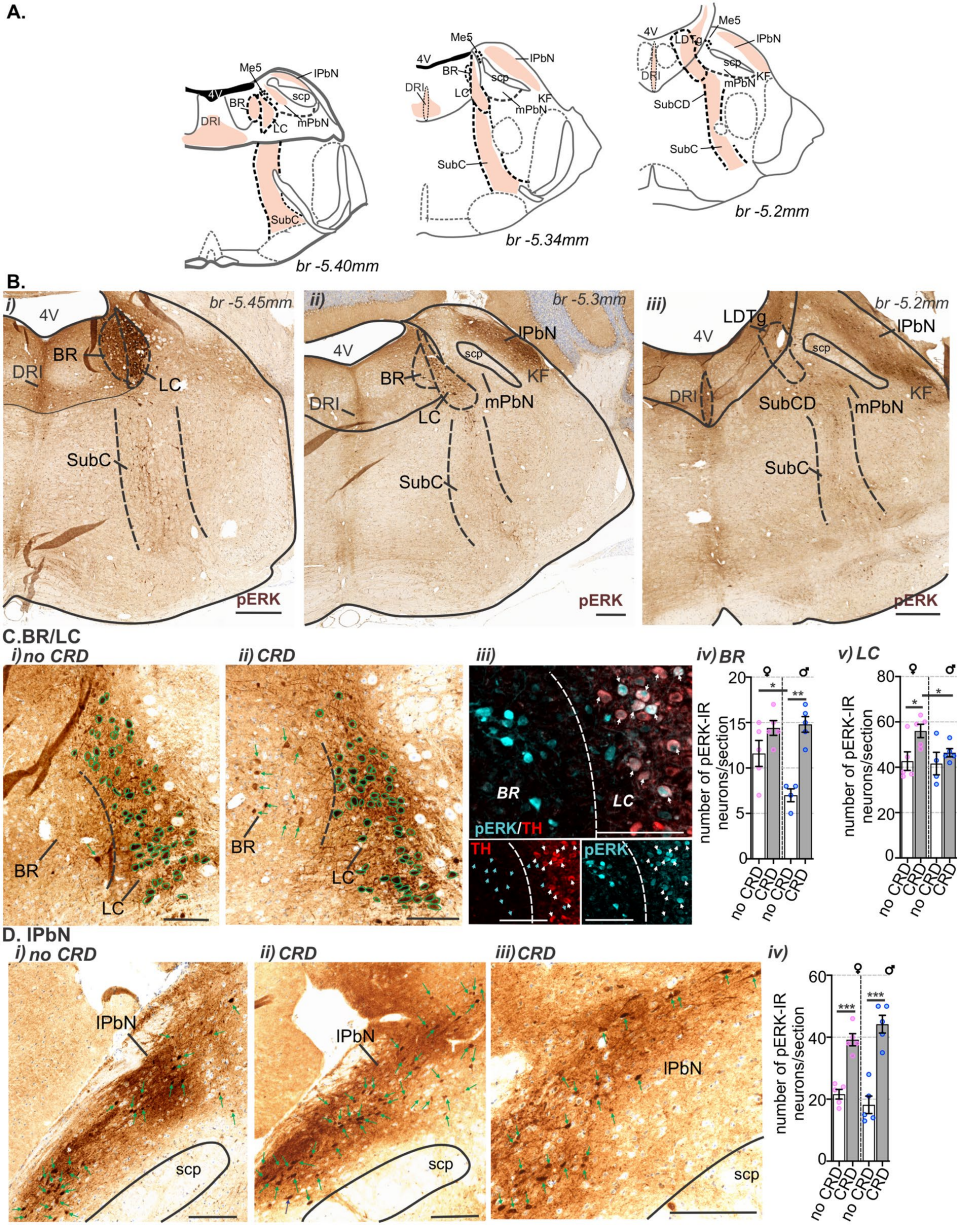
Distribution of pERK‐immunoreactive (pERK‐IR) neurons in the pons following in vivo colorectal distension (CRD). (A) Schematic representation of the pons (Paxinos and Franklin 2001) summarizing the distribution (regions indicated by orange shading). (B) Low magnification photomicrographs of (i) caudal, (ii) intermediate, and (iii) rostral pontine sections showing the overall distribution of pERK‐IR neurons (brown) following in vivo CRD at noxious pressures. pERK‐IR neurons were present within the (i, ii) Barrington's nucleus (BR), locus coeruleus (LC), intermediate reticular tracts (IRt), the (ii, iii) subcoeruleus (SubC), lateral parabrachial nucleus (lPbN), and (i–iii) in the dorsal raphe nuclei (interfascicular; DRI) and the (iii) laterodorsal tegmental nucleus (LDTg). Scale bars: 500 μm. (C, D) High magnification photomicrographs showing the distribution of pERK‐IR neurons (cells, indicated by green outline or green arrows) within the (C) BR and LC (BR/LC) and (D) lPbN from mice that underwent (i) no CRD or (ii, iii) noxious CRD (CRD). (Ciii) Photomicrograph of the BR/LC immunolabeled for pERK (cyan) and tyrosine hydroxylase (TH, red); pERK/TH co‐labeled cells are indicated by white arrows. Scale bars = 100 μm. Quantification comparing the number of pERK‐IR neurons between female (♀, pink data points) and male (♂, blue data points) mice that underwent no CRD (white bar) or noxious CRD (gray bar) within the (Civ) BR, (Cv) LC, and the (Dv) lPbN. *p < 0.05, **p < 0.01, ***p < 0.001, determined by unpaired t‐tests. Data points represent the mean number of pERK‐IR neurons/section of individual mice, gained from 2 to 4 sections/mouse from No CRD: *N* = 5F:5M mice (5F:4M for BR and LC) and CRD: 5F:5M mice. Error bars represent the standard error of the mean. Abbreviations: 4 V, Fourth ventricle; br, bregma; KF, Kölliker‐Fuse nucleus; Me5, mesencephalic trigeminal nucleus; mPbN, medial parabrachial nuclei; scp, superior cerebellar peduncle; SubCD, subcoeruleus dorsal.

In the pons (Figure 10), similar to the distribution of H129‐EGFP+ cells, pERK‐IR neurons were localized within BR and the LC (Figure 10A,Bi,Bii,C), the IRt and subC (Figure 10A,Bi–iii) and the dorsal aspect of the lPbN (Figure 10A,Bii,Biii,D). In the BR (Figure 10C), relative to no CRD controls (Figure 10Ci), the number of pERK‐IR neurons was greater following noxious CRD (Figure 10Cii). This increase was significant in male mice but not female mice (Figure 10Civ). Conversely, in the LC (Figure 10C), relative to no CRD controls (Figure 10Ci), the number of pERK‐IR neurons increased following noxious CRD (Figure 10Cii) significantly within female in mice but not male mice (Figure 10Cv). In the lPbN (Figure 10D), relative to no CRD controls (Figure 10Di), the number of pERK‐IR neurons was significantly increased following noxious CRD (Figure 10Dii,Diii) in male and female mice (Figure 10Div).

**FIGURE 11:**
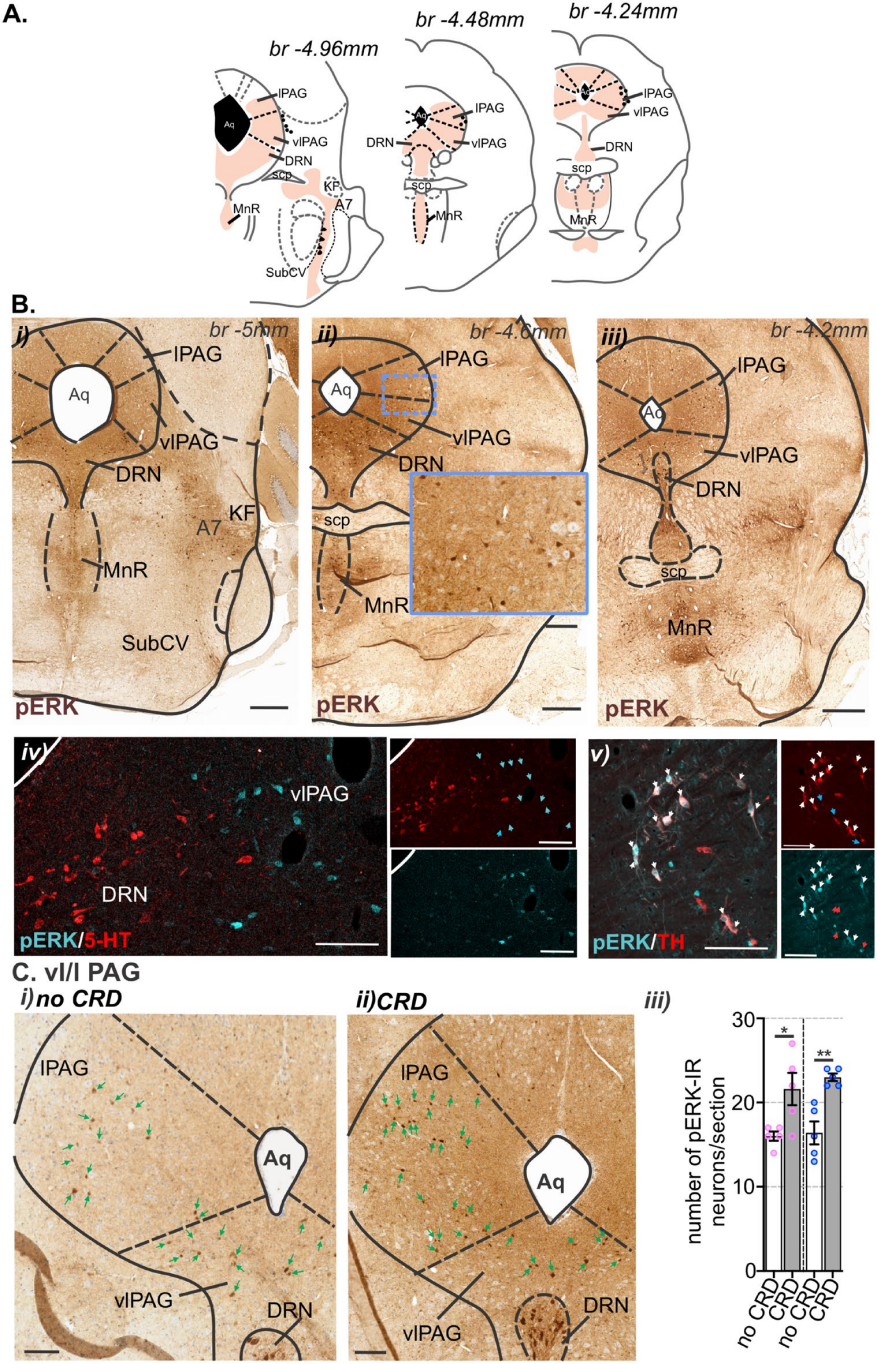
Distribution of pERK‐immunoreactive (pERK‐IR) neurons in the midbrain following in vivo colorectal distension (CRD). (A) Schematic representation of the caudal midbrain (Paxinos and Franklin 2001) summarizing the distribution (regions indicated by orange shading). (Bi–iii) Low magnification photomicrographs of caudal midbrain sections showing the overall distribution of pERK‐IR neurons (brown) following in vivo CRD at noxious pressures. pERK‐IR neurons were present within the (i–iii) periaqueductal gray (PAG), in the lateral (lPAG) and ventrolateral (vlPAG) sub‐regions, in addition to the dorsal raphe nuclei (DRN) and the median raphe nucleus (MnR). (ii) The inset image (scale bar = 100 μm) corresponds to the region within the dotted boxed area. Scale bars = 500 μm. (Biv) High magnification photomicrographs of the vlPAG/DRN region immunolabeled for pERK (cyan) and serotonin (5‐HT, red); pERK labeled cells are indicated by cyan arrows. Scale bars: 100 μm. (Bv) High magnification photomicrographs of the A7 noradrenaline cellular region immunolabeled for pERK (cyan, cyan arrows) and tyrosine hydroxylase (TH, red, red arrows); pERK/TH co‐labeled cells are indicated by white arrows. Scale bars: 100 μm. (C) High magnification photomicrographs of the vl/lPAG region showing the distribution of pERK‐IR neurons (cells, indicated by green arrows) within mice that underwent (i) no CRD or (ii) noxious CRD (CRD). Scale bars: 100 μm. (iii) Quantification comparing the number of pERK‐IR neurons per section within the vl/lPAG between female (♀, pink data points) and male (♂, blue data points) mice that underwent no CRD (white bar) or noxious CRD (gray bar). *p < 0.05, **p < 0.01 determined by unpaired t‐tests. Data points represent the mean number of pERK‐IR neurons/section of individual mice, gained from 2 to 3 sections/mouse from no CRD: *N* = 5F:5M mice and CRD: 5F:5M mice. Error bars represent the standard error of the mean. Abbreviations: Aq, aqueduct; br, bregma; KF, Kölliker‐Fuse nucleus; Me5, mesencephalic trigeminal nucleus; scp, superior cerebellar peduncle; SubCV, subcoeruleus ventral.

In the midbrain (Figure 11), aligning with the distribution of H129‐EGFP+ cells, pERK‐IR neurons were observed within the ventrolateral and lateral subregions of the PAG (Figure 11A,Bi–iii,Biv,C), the DRN (Figure 11A,Bi–iii), the ventral SubC (Figure 11A,Bi), and within the A7 (Figure 11A,Bi). Similar to H129‐EGFP+ cells, pERK‐IR neurons in the A7 co‐labeled for TH (Figure 11Bv). In the PAG compared to no CRD (Figure 11Ci), noxious CRD (Figure 11Cii) evoked a significant increase in the number of pERK‐IR neurons (Figure 11Ciii), which was equal in female and male mice.

**FIGURE 12:**
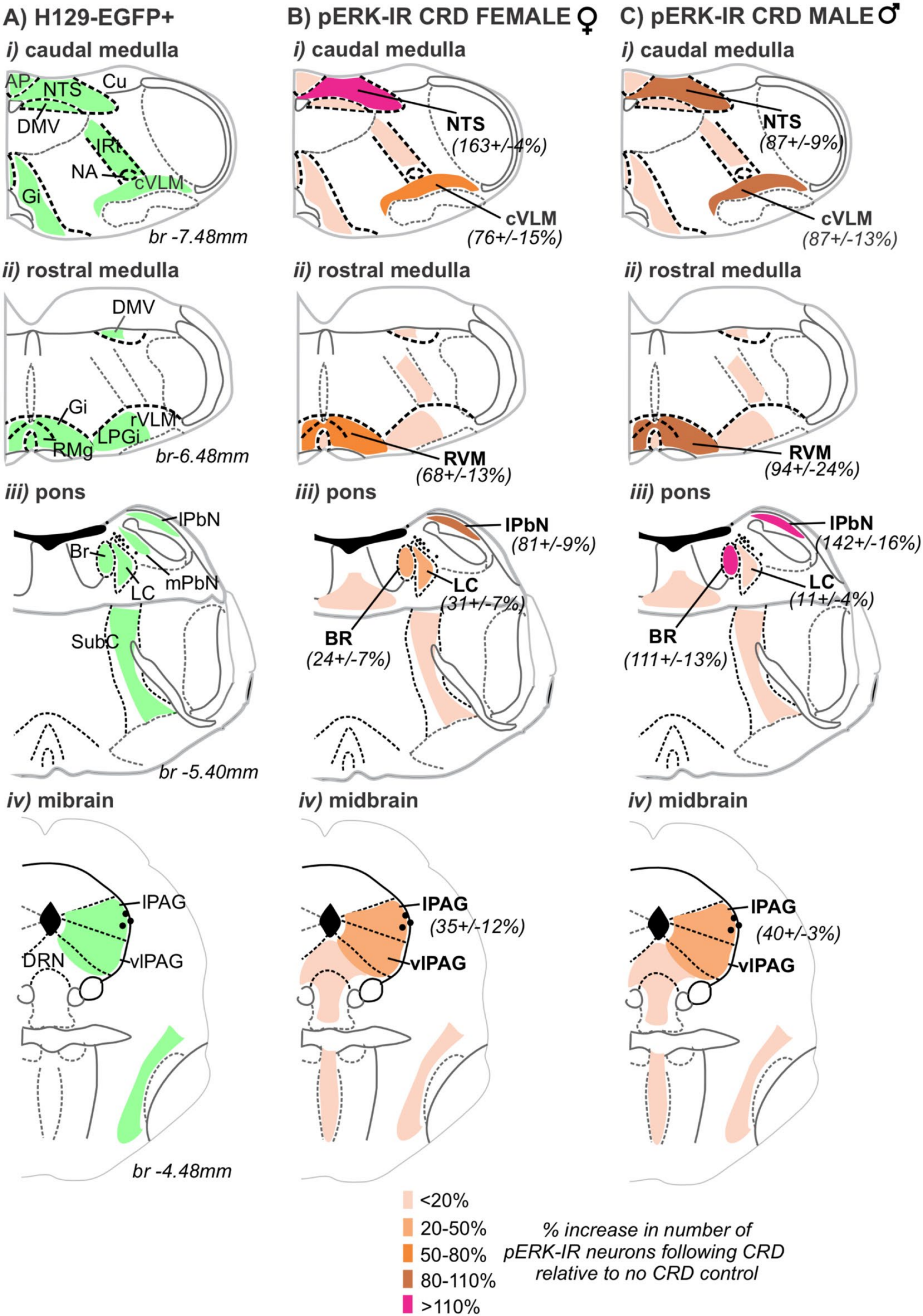
Summary diagram comparing the distribution of H129‐EGFP+ cells following colorectal wall inoculation and pERK‐IR neurons following in vivo noxious colorectal distension (CRD). Schematic (Paxinos and Franklin 2001) comparing the distribution (shaded regions) of (A) H129‐EGFP+ in tissue collected 120 h after colorectal inoculation and (B, C) pERK‐IR neurons following CRD, and the percent increase (expressed as the mean plus or minus the standard error of the mean) in the number of pERK‐IR neurons evoked by CRD, relative to no CRD (highlighted by density in shading) in (B) female and (C) male mice.

In summary (Figure 12), in many of the brainstem structures we observed H129‐EGFP+ cells (Figure 12); we also observed an increase in the number of pERK‐IR neurons evoked by in vivo CRD, relative to no CRD controls (Figure 12B,C). The percentage increase in the number of pERK‐IR neurons relative to the number of pERK‐IR neurons following no CRD differed between female (Figure 12B) and male (Figure 12C) mice noticeably in the BR, LC, and lPbN.

### 3.3 The Effect of Removing DRG at Either TL or LS Spinal Levels on CRD Evoked Neuronal Activation in the Brainstem

**FIGURE 13:**
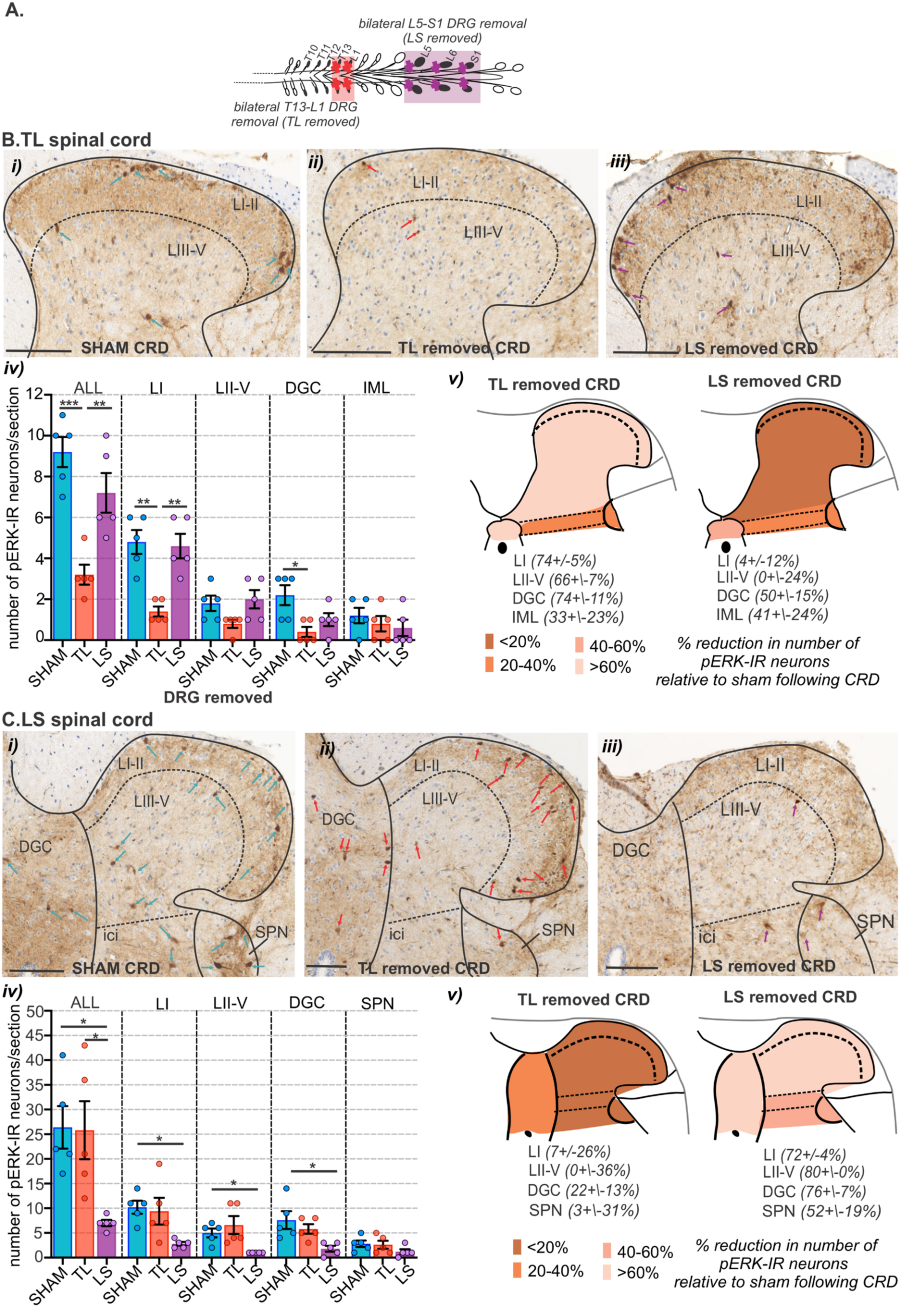
The effect of removing thoracolumbar (TL, T13‐L1 DRG) and lumbosacral (LS, L5‐S1) DRG on the number of pERK‐IR neurons within the spinal cord following in vivo noxious colorectal distension (CRD). (A) Schematic illustration showing the spinal levels at which dorsal root ganglia (DRG) were surgically removed in experimental groups TL removal (T13‐L1 DRG, red box) and LS removal (L5‐S1 DRG, purple box). The effect of either TL or LS DRG removal on neuronal activation (pERK‐IR neurons) evoked by noxious CRD was compared to that in sham mice (that underwent the same surgical laminectomy procedure without DRG removal). (B) Photomicrographs showing the distribution of pERK‐IR neurons (pERK, brown, indicated by arrows) within the TL spinal cord dorsal horn following noxious CRD in (i) sham mice (blue arrows) and mice with (ii) TL (red arrows) or (iii) LS (purple arrows) DRG removed. Scale bars: 100 μm. (iv) Quantification of the mean number of pERK‐IR neurons in the TL spinal cord dorsal horn all laminae (ALL), lamina I (LI), laminae II–V (LII–V), dorsal gray commissure (DGC), and the intermediolateral nuclei (IML) compared between sham (blue bar), TL (red bar), and LS (purple bar) removed mice. *p < 0.05, **p < 0.01, and ***p < 0.001 determined by one‐way ANOVA (parametric data) with Bonferroni multiple comparison tests. Individual data points represent the mean number of pERK‐IR neurons per section of individual mice, gained from 5 sections/mouse, gained *N* = 5 female mice/group. Error bars represent the standard error of the mean. (v) Schematic illustration of the TL spinal cord dorsal horn summarizing the reduction in the number of pERK‐IR neurons as a percent (%) of sham mice in the LI, LII–V, DGC, and IML of TL and LS removed mice. (C) Photomicrographs showing the distribution of pERK‐IR neurons (pERK, brown, indicated by arrows) within the LS spinal cord dorsal horn following noxious CRD in (i) sham mice (blue arrows) and mice with (ii) TL (red arrows) or (iii) LS (purple arrows) DRG removed. Scale bars: 100 μm. (iv) Quantification of the mean number of pERK‐IR neurons in the LS spinal cord dorsal horn all laminae (ALL), lamina I (LI), laminae II–V (LII–V), dorsal gray commissure (DGC) and the sacral parasympathetic nuclei (SPN) compared between sham (blue bar), TL (red bar) and LS (purple bar) removed mice. *p < 0.05, determined by one‐way ANOVA (parametric data) with Bonferroni multiple comparison tests. Individual data points represent the mean number of pERK‐IR neurons per section of individual mice, gained from 5 sections/mouse, gained from *N* = 5 female mice/group. Error bars represent the standard error of the mean. (v) Schematic illustration of the LS spinal cord dorsal horn summarizing the reduction in the number of pERK‐IR neurons as a percent (%) of sham mice in the LI, LII–V, DGC, and SPN of TL and LS removed mice. The percent reduction is expressed as a mean percent plus or minus the standard error of the mean. Abbreviations: DGC, dorsal gray commissure; ici, intercalated nuclei; IML, intermediolateral nuclei; LI, lamina I; LII, lamina 2; LIII, lamina 3; LIV, lamina 4; LV, lamina 5; SPN, sacral parasympathetic nuclei.

**FIGURE 14:**
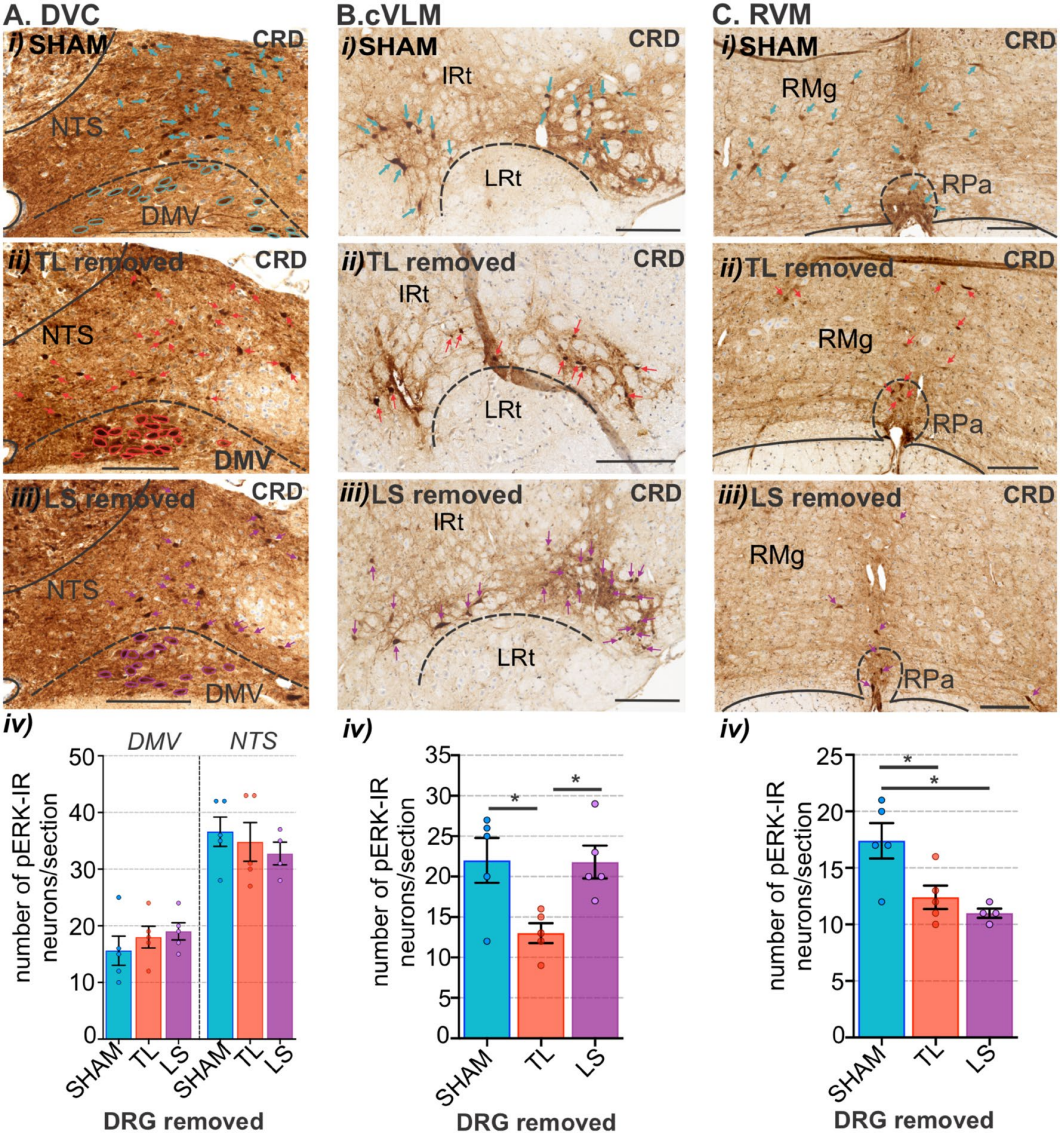
The effect of removing thoracolumbar (TL, T13‐L1 DRG) and lumbosacral (LS, L5‐S1) DRG on the number of pERK‐IR neurons within the medulla following in vivo noxious colorectal distension (CRD). Photomicrographs showing pERK‐IR neurons (pERK, brown, indicated by arrows) in (A) caudal dorsal vagal complex (DVC), (B) caudal ventrolateral medulla (cVLM), and (C) rostral ventromedial medulla (RVM) following noxious CRD in (i) sham mice (blue arrows) and mice with (ii) TL (red arrows) or (iii) LS (purple arrows) DRG removed. Scale bars: 100 μm. Quantification comparing the number of pERK‐IR neurons between sham (blue bars), TL (red bars), and LS (purple bars) removed mice in the (Aiv) dorsal vagal motor nuclei (DMV) and nucleus of the solitary tract (NTS), (Biv) cVLM (inclusive of the A1 region ventral to the intermediate reticular (IRt) and dorsal to the lateral reticular nucleus (LRt)) and (Civ) RVM (inclusive of the raphe pallidus nucleus (RPa) and raphe magnus nucleus (RMg)). *p < 0.05, determined by a one‐way ANOVA with Bonferroni multiple comparison tests (parametric data). Data points represent the mean number of pERK‐IR neurons per section of individual mice, gained from 1 to 3 sections/mouse (*N* = 5 mice/group; RVM LS removed *N* = 4), Error bars represent the standard error of the mean.

**FIGURE 15:**
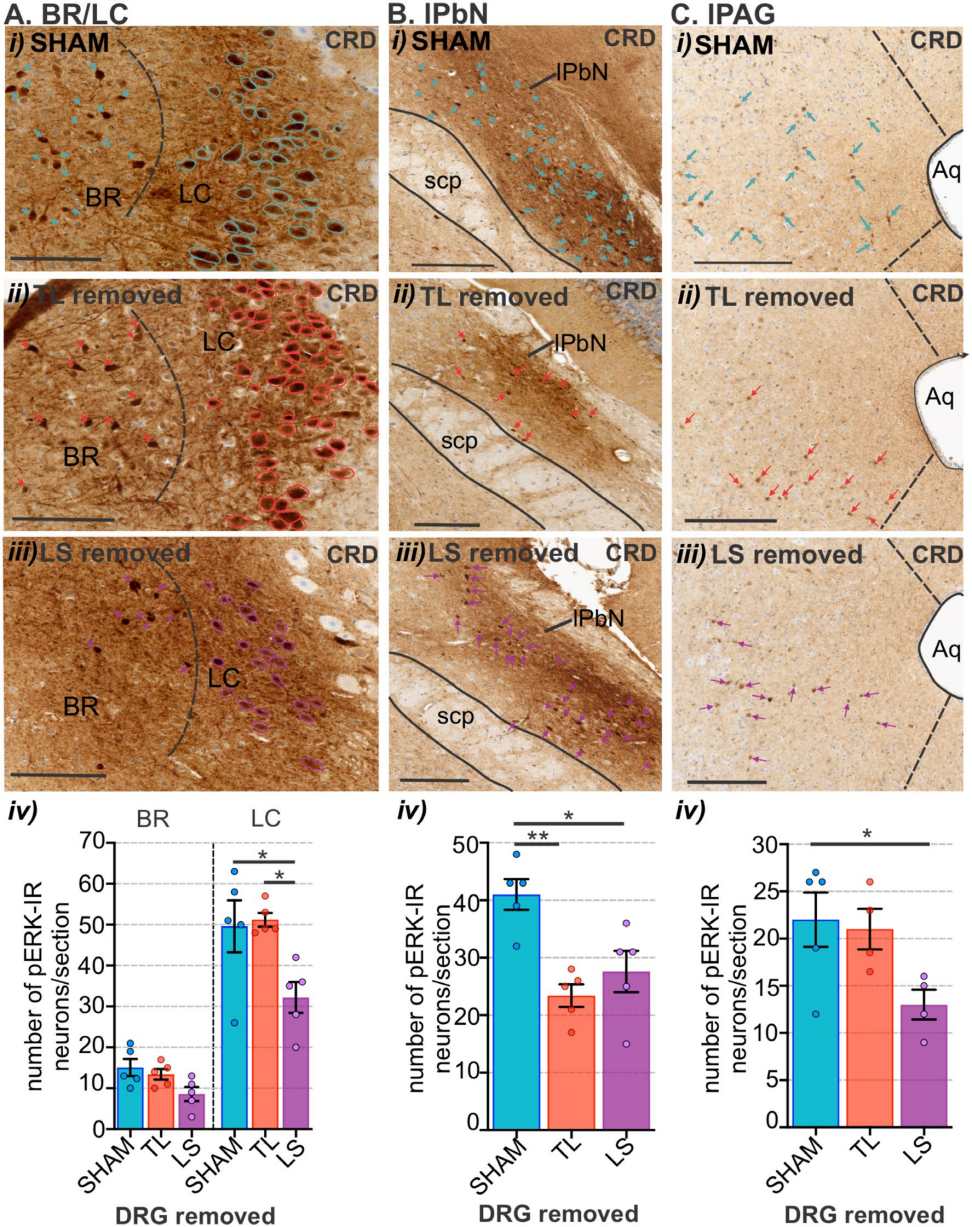
The effect of removing thoracolumbar (TL, T13‐L1 DRG) and lumbosacral (LS, L5‐S1) DRG on the number of pERK‐IR neurons within the pons and midbrain following in vivo noxious colorectal distension (CRD). Photomicrographs showing pERK‐IR neurons (pERK, brown, indicated by outline or arrows) in the pontine (A) Barrington's nucleus (BR) and locus coeruleus (LC) and (B) lateral parabrachial nucleus (lPbN), and the caudal midbrain (C) periaqueductal gray (PAG), lateral (lPAG) sub‐region, following noxious CRD in (i) sham mice (blue arrows or outline) and mice with (ii) TL (red arrows or outline) or (iii) LS (purple arrows or outline) DRG removal. Scale bars: 100 μm. Quantification comparing the number of pERK‐IR neurons in the (Aiv) Br and LC, (Biv) lPbN, and (Civ) lPAG between sham (blue), TL (red), and LS (purple) removal mice. *p < 0.05, **p < 0.01, determined by a one‐way ANOVA with Bonferroni multiple comparison tests (parametric data). Data points represent the mean number of pERK‐IR neurons per section of individual mice *N* = 5 mice/group (PAG TL removed, and LS removed *N* = 4), gained from 1 to 3 sections/mouse. Error bars represent the standard error of the mean. Abbreviations: Aq = aqueduct and scp = superior cerebellar peduncle.

We then assessed the degree to which CRD‐evoked neuronal activation in the brainstem was shaped by either of the sensory afferent pathways ascending from the colorectum. To do this we used bilateral removal of the TL (T13‐L1) or LS (L5‐S1) DRG (Figure 13A) and assessed the number and distribution of pERK‐IR neurons in the spinal cord (Figure 13) and brainstem (Figure 14 and Figure 15) relative to sham surgery (no DRG removal). Removal of DRGs resulted in a reduction in the number of pERK‐IR neurons evoked by CRD within the corresponding TL (Figure 13B) and LS (Figure 13C) spinal cord, as previously reported (Kyloh et al. 2022). In the TL dorsal horn, the number of pERK‐IR neurons relative to sham (Figure 13Bi) was significantly reduced by TL DRG removal (Figure 13Bii) but not by LS DRG removal (Figure 13Biii,Biv). This reduction was most evident in the dorsal horn LI (Figure 13Biv,Bv). In the LS dorsal horn, the number of pERK‐IR neurons relative to sham (Figure 13Ci) was not significantly reduced by removal of TL DRG (Figure 13Cii) but by LS DRG removal (Figure 13Ciii,Civ). This reduction was significant in LI, LII–V, and the DGC (Figure 13Civ,Cv). In the medulla DVC, relative to sham (Figure 14Ai), DRG removal at either spinal level, had no effect on the number of pERK‐IR neurons evoked by CRD within the DMV or the NTS (Figure 14Aii–iv). Relative to the number of CRD‐evoked pERK‐IR neurons in sham mice (Figure 14Bi,Ci,Ai,Bi,Ci), TL DRG removal significantly reduced pERK‐IR neurons within the cVLM (Figure 14Bii,Biv), the RVM (Figure 14Cii,Civ) and the lPbN (Figure 15Bii,Biv), but not the BR and LC (Figure 15Aii,Aiv) nor the PAG (Figure 15Cii,Civ). LS DRG removal did not reduce the number of CRD‐evoked pERK‐IR neurons in the cVLM (Figure 14Biii,Biv) but did significantly reduce the number of pERK‐IR neurons in the RVM (Figure 14Ciii,Civ), the BR and the LC (Figure 15Aiii,Aiv), the lPbN (Figure 15Biii,Biv), and the PAG (Figure 15Ciii,Civ).

**FIGURE 16:**
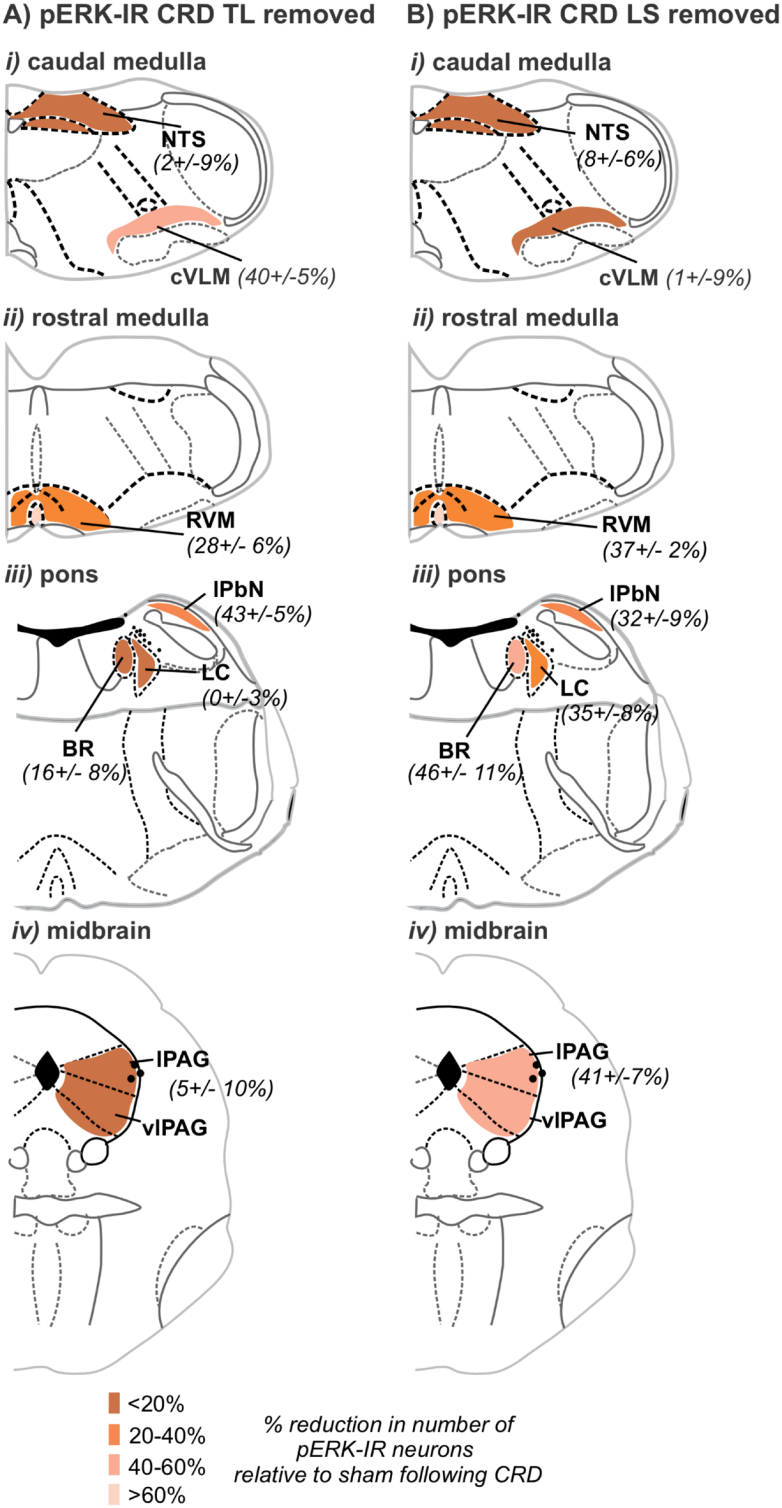
Summary diagram of the effect DRG removal had on the number of pERK‐IR neurons in the brainstem evoked by in vivo noxious colorectal distension (CRD). Schematic (Paxinos and Franklin 2001) illustration of the brainstem regions summarizing the reduction in the number of pERK‐IR neurons evoked by CRD relative to sham mice (expressed as a mean percent (%) reduction plus or minus the standard error of the mean) following (A) TL DRG removal or (B) LS DRG removal. The reduction in pERK‐IR number is highlighted by the density of shading.

In summary (Figure 16), removal of TL DRG (splanchnic afferent pathway) reduced CRD‐evoked neuronal activation in the cVLM, the RVM, and the lPbN (Figure 16A). Removal of the LS DRG (pelvic afferent pathway) resulted in a reduction of CRD‐evoked neuronal activation within the RVM, LC, BR, lPbN, and the PAG (Figure 16B). As a percentage of the neuronal activation in sham surgery mice, the reduction of pERK‐IR neurons in the lPbN and RVM was equivalent between TL or LS DRG removal (Figure 16A,B). While the reduction of CRD‐evoked neuronal activation in the cVLM relative to sham was unique to TL DRG removal (Figure 16A), the reduction in CRD‐evoked neuronal activation in the BR, LC, and PAG was unique to LS DRG removal (Figure 16B).

## 4 Discussion

This study identified the brainstem structures relevant to colorectal sensory‐motor processing in the mouse. We specifically compared the relative contribution of ascending sensory signaling via the TL and LS spinal cord to directing where colorectal sensory‐motor processing occurs within brainstem structures. First, we tracked over time where H129‐EGFP+ transneuronal labeling from the distal colon occurred within the spinal pathways and the brainstem. Confirming their functional relevance to colorectal processing, we then showed that noxious CRD‐evoked increased neuronal activation, relative to no CRD, in many of the H129‐EGFP+ labeled brainstem structures. We then showed that the removal of DRG at spinal levels known to contain splanchnic and pelvic colorectal afferent neurons reduced CRD evoked neuronal activation in overlapping and unique brainstem regions. Collectively, this data provides new insight into how the dual spinal afferent innervation of the colorectum differentially shapes where colorectal sensory‐motor information is relayed and integrated within brainstem circuits relevant to sensory and motor aspects of colorectal function.

We have previously shown that colorectal spinal sensory afferent fibers project within discrete regions of the spinal cord dorsal horn using retrograde tracing from the colorectum in the mouse (Harrington et al. 2019; Wang et al. 2023). In the TL spinal cord, colorectal afferent fibers project within the dorsal horn LI, LIV–V, DGC, and the IML, and within dorsal horn LI, LIII–V, DGC, and the SPN in the LS spinal cord. The differences we observed in the distribution and density of H129‐EGFP+ cells between the TL and LS dorsal horn align with these previous findings. Viral‐labeled cells first appeared in the dorsal horn LI in both the TL and LS spinal cord. In the TL dorsal horn 96 h post‐inoculation, viral‐labeled cells were observed in LIII–V and DGC. While in the LS dorsal horn, viral‐labeled cells were denser and more widespread than those of the TL dorsal horn, evident in LII–LV, the DGC, and within the SPN. We have also observed colorectal afferent fibers projecting into these laminae (Harrington et al. 2019; Wang et al. 2023). In tissue collected 120 h post‐inoculation, viral labeling appeared fragmented, particularly in the LS dorsal horn, possibly indicative of an earlier viral infection that has moved on. The distribution of H129‐EGFP+ cells was also similar to the distribution of pERK‐IR neurons we have observed in the TL and LS spinal cord in previous studies and in sham surgery mice in the current study (Grundy et al. 2018; Harrington et al. 2019; Kyloh et al. 2022; Wang et al. 2024). Collectively, these findings, in addition to the labeling of DRG, indicate that the H129‐EGFP+ labeling we observed in the spinal cord was a product of input from colorectal sensory afferent fibers.

In addition to these sensory‐processing laminae of the dorsal horn, H129‐EGFP+ cells were evident in autonomic nuclei of the thoracic IML and the sacral SPN, where they were associated with labeling in ventrolateral tracts in the spinal cord white matter. This labeling is possibly a product of off‐target sites (non‐colonic) exposed to the virus. Against this, however, we did not observe H129‐EGFP+ cells in spinal cord segments (L3‐L4) that are known not to receive colorectal afferent input. Moreover, we did not see an abundance of labeling in the NTS that would be expected if the virus traveled off‐target into the proximal colon and into vagal sensory pathways. A more likely explanation is that this labeling was a consequence of HSV‐1 H129 producing first‐order retrograde infection of central autonomic sympathetic (IML) and parasympathetic (SPN) preganglionic motor neurons that project directly, or indirectly via prevertebral ganglia, to inoculation sites within the colorectum. As evidence of this, we have previously shown populations of SPN neurons are retrograde labeled directly from the colorectum (Harrington et al. 2019; Wang et al. 2024). Studies using dual HSV‐1 H129 and PRV labeling from the stomach and small intestine of the rat illustrate that both produce retrograde infection of central autonomic nuclei (Rinaman and Schwartz 2004; Parker et al. 2020). Moreover, they provide neuroanatomical evidence for interconnectivity between sensory and autonomic circuits within the gut‐brain axis throughout the entire brain, in addition to sensory and autonomic information being processed by separate neuronal populations within the same brainstem structures (Parker et al. 2020). This highlights an important caveat when interpreting the origins of H129‐EGFP+ labeling within the brainstem we observed in the present study. Such labeling could be a product of both the sensory and autonomic pathways connected to the colorectum that ascend via the spinal cord. Likewise, the neuronal activation evoked by CRD over and above that of no CRD would be a summation of activity within sensory and autonomic pathways involved in affective colorectal sensory‐motor responses. Importantly, our data from the lesioning of DRG identifies how much of the CRD‐evoked neuronal activation within the brainstem is a consequence of the ascending sensory spinal pathways.

The distribution pattern of H129‐EGFP+ cells we observed in the brainstem aligned with findings from the previous studies using PRV transneuronal tracing from the rat colorectum (Pavcovich et al. 1998; Valentino et al. 2000; Vizzard et al. 2000; Rouzade‐Dominguez et al. 2003; He et al. 2018). In the brainstem, H129‐EGFP+ cells were observed within structures known to receive abundant sensory ascending input from the spinal cord dorsal horn, namely reticular formations within the caudal and rostral VLM and RVM, the lPbN, and the PAG (Lima et al. 1991; Wang et al. 1999; Polgar et al. 2010; Todd 2010; Martins and Tavares 2017; Wercberger and Basbaum 2019; Gu et al. 2023). H129‐EGFP+ cells were also observed in brainstem structures that are traditionally thought to receive relatively little direct ascending sensory input from the spinal cord yet are origins of dense descending output to the spinal cord, such as the BR, LC, and RVM (Cedarbaum and Aghajanian 1978; Ding et al. 1997; Wang et al. 1999; Samuels and Szabadi 2008; Peng et al. 2023). The studies using dual HSV‐1 H129 and PRV‐labeling illustrate that unlike PRV, HSV‐1 transport through the spinal cord and brain occurs predominantly in the anterograde (ascending) direction via their synaptic output (Rinaman and Schwartz 2004; Parker et al. 2020). Transport of HSV‐1 in the retrograde direction from infected neurons into cell bodies of their synaptic inputs descending from the brain was found to be limited (Rinaman and Schwartz 2004). This distinction has particular relevance to our study, as we wanted to identify brainstem structures targeted by ascending pathways and limit the potential for retrograde labeling of brainstem structures via their descending projections onto HSV‐1 H129‐infected spinal cord neurons. While in our study this mode of transport cannot be completely excluded, a discrepancy between H129‐EGFP+ and pERK‐IR labeling of TH‐IR catecholamine A1 neurons within the cVLM may support a preferential anterograde (ascending) transport of HSV‐1 H129. Populations of A1 catecholaminergic neurons activated by nociceptive stimuli have descending outputs in the spinal cord, but do not receive direct input from the spinal cord (Tavares and Lima 2002; Gu et al. 2023). We observed many H129‐EGFP+‐labeled cells in the cVLM; however, these were not TH‐IR catecholamine neurons. This is despite observing populations of pERK‐IR within the cVLM that were A1 catecholaminergic neurons.

Sympathetic preganglionic motor neurons of the IML in the thoracic spinal cord relay into noradrenergic cell groups in the midbrain A7, LC, and A5 in the pons and the cVLM (A1/C1 neurons) and RVLM (Shahar and Palkovits 2007; Delbono et al. 2022). Populations of IML neurons also project to the NTS and the Kolliker‐Fuse (KF) nucleus of the PbN complex (Cedarbaum and Aghajanian 1978). Parasympathetic preganglionic neurons in the SPN project into the BR, LC, and lPbN (Ding et al. 1997; Wang et al. 1999). As such, the H129‐EGFP‐labeling and the CRD‐evoked pERK neuronal activation we observed in these structures can also be attributed to ascending output from these autonomic pathways, in particular, the H129‐EGFP+ TH‐IR neurons A7 in the midbrain. However, as mentioned above, the TH‐IR A1/C1 neurons of the cVLM were not H129‐EGFP+, which would have been expected if ascending input was solely received via the IML. We also observed H129‐EGFP+ cells; moreover, DRG removal reduced pERK‐IR neurons in the dorsal aspect of the lPbN that are more relevant to sensory processing, rather than the KF. We did observe pERK‐IR TH‐IR neurons in the A1/C1, A7, and in the KF. Moreover, when looking at the reduction in the BR and cVLM by DRG removal collectively, it was comparatively less than the overall increase in neuronal activation evoked by CRD relative to no CRD controls. This indicates that signaling within autonomic pathways contributed to CRD‐evoked activation within these structures and aligns with their respective roles as key central autonomic nuclei coordinating defecation and sympathetic tone.

Sensory information relevant to nociception is relayed from the spinal cord into the brainstem primarily by populations of neurons within the dorsal horn LI (Wercberger and Basbaum 2019; Peirs et al. 2020). These neurons relay into reticular formations within the cVLM, the NTS, the lPbN, and the PAG (Todd 2010; Cameron et al. 2015; Martins and Tavares 2017; Wercberger and Basbaum 2019). Populations of dorsal horn neurons relevant to transmitting a wider range of sensory information, a mix of nociceptive and non‐nociceptive, are scattered throughout laminae III–VI and the DGC (Wercberger and Basbaum 2019). These neurons project widely within the brainstem, into the NTS, the medullary and pontine reticular formation, the lPbN, and the PAG (Todd 2010; Martins and Tavares 2017; Wercberger and Basbaum 2019). Reflecting on this knowledge, observing H129‐EGFP+ cells distributed within laminae LI and LIII–V of the TL dorsal horn aligns with splanchnic colorectal afferents relaying into dorsal horn circuits primarily concerned with processing and relaying nociceptive information (Harrington et al. 2019; Wang et al. 2024). Illustrated by the removal of TL DRG, this supraspinal output likely contributes to viral labeling in the reticulum of the cVLM, RVM, and the lPbN. Such findings are interesting in the context of colorectal hypersensitivity being linked to heightened activity within the TL dorsal horn and increased output to the lPbN in models of colitis (Traub 2000; Harrington et al. 2012). Moreover, heightened activity within the cVLM is also evident post‐colitis, which may be mediated by increased activity within the TL spinal cord (Lyubashina et al. 2019). Conversely, in the LS spinal cord dorsal horn, H129‐EGFP+ cells were widely distributed within laminae LI–LV, DGC, and SPN. This distribution pattern agrees with our previous findings of pelvic colorectal afferent fibers relaying a mixture of sensory information into dorsal horn circuits concerned with the supraspinal relay of nociceptive and non‐nociceptive sensory and interoceptive information, interneuronal processing, and parasympathetic‐mediated reflexes (Harrington et al. 2019; Wang et al. 2024). The effect of LS DRG removal illustrates that information from pelvic colorectal afferents is relayed into multiple brainstem structures (RVM, lPbN, BR, LC, and the PAG) that are involved in a diverse range of sensory and autonomic functions (Liu et al. 2007; Naitou et al. 2018; Nakamori et al. 2018; Lyubashina et al. 2022; Qi et al. 2022).

Removal of either TL or LS DRG significantly reduced CRD‐evoked neuronal activation within the lPbN. As a major relay nucleus, the lPbN distributes incoming sensory information widely within cortico‐limbic and brainstem structures that shape pain behavior, modulation, and integration with autonomic responses (Roeder et al. 2016; Chiang et al. 2020). The lPbN receives input from the spinal cord neurons distributed within the dorsal horn LI and LIII–IV and the SPN (Choi et al. 2020; Browne et al. 2021). The effect we observed of TL DRG removal on the lPbN aligns with our recent findings of colorectal splanchnic afferent fibers in apposition to lPbN‐projecting neurons within LI and that a large proportion (63%) of neurons activated by noxious CRD within LI of the TL spinal cord project to the lPbN (Wang et al. 2024). In the LS spinal cord, whilst lPbN‐projection neurons make up a relatively small proportion of the CRD‐responsive neurons in the dorsal horn LI (13%), lPbN‐projecting neurons responsive to CRD have also been observed in the SPN, DGC, and lateral gray matter (Murphy et al. 2009; Nishida et al. 2022; Wang et al. 2024). These differences indicate that the lPbN receives different qualities of colorectal information from the TL spinal cord than the LS spinal cord. There is evidence of this, from the LS spinal cord at least, in an optogenetic study showing that populations of lPbN‐projecting neurons within the DGC of the sacral spinal cord are involved in colitis‐evoked aversion responses but not visceromotor responses (Qi et al. 2022). The neurocircuits of the lPbN receiving differential colorectal input from the TL and LS spinal cord warrant further investigation, in particular assessing if they relay into disparate cortico‐limbic circuits that were not the focus of this study to influence pain behaviors.

TL and LS DRG removal reduced the number of RVM neurons activated by noxious CRD equally. In the case of the TL pathway, this may be mediated via the lPbN, as the RVM primarily receives ascending nociceptive information via the lPbN (Peng et al. 2023). Under physiological conditions, and when exposed to acute pain stimuli, this lPbN‐RVM circuit triggers acute hyperalgesia (Peng et al. 2023). As for the LS pathway, the reduction in the RVM may not solely be linked to reduced output from the lPbN but may also be mediated by its connectivity with the LC and PAG. The activity was also reduced by LS DRG removal. The LC and RVM are key structures of the descending pain modulatory system linked to both pro‐ and anti‐nociception (Peng et al. 2023). They each have roles in the spinal transmission of visceral pain via their descending outputs to the LS spinal cord that inhibit or facilitate neuronal response to CRD (Liu et al. 2008; Lubejko et al. 2024). Stimulation of the PAG also inhibits CRD‐evoked activity within the LS spinal cord dorsal horn LI (Okada et al. 1999; Lyubashina et al. 2022). This is proposed to be mediated by reciprocal loops between the sacral spinal cord and the PAG, via the RVM, LC, and BR. The ventrolateral and lateral subregions of the PAG, in which we observed H129‐EGFP+ cells and neurons activated by CRD, receive direct input from neurons in the sacral spinal cord dorsal horn (LI, LV, and the lateral spinal nucleus) (Keay et al. 1997). Input from these dorsal horn neurons recruits PAG output to the RVM and the LC, which in turn feeds back to the dorsal horn. Such circuitry has been shown to generate defensive responses to pain, and impairment of this circuitry is evident in models of colitis‐induced colorectal hypersensitivity (Lyubashina et al. 2022). Our findings highlight the importance of targeting pelvic afferent fibers to correct such impairment. Reciprocal circuits between the LS spinal cord and the LC and BR, or their recruitment via the PAG, are also implicated in regulating micturition and defecation reflexes (Ding et al. 1997; Pavcovich et al. 1998; Nakamori et al. 2019). Collectively, our findings align with colorectal pelvic afferents having a predominant role of relaying sensory information into brainstem descending pain modulatory systems and autonomic nuclei regulating defecation reflexes, in addition to nuclei involved in ascending sensory relay. To confirm exactly where the direct input from the LS dorsal horn occurs within these networks, the proportions of neurons in the LS dorsal horn projecting to the lPbN, BR, LC, or PAG activated by colonic stimulation, and vice versa, are required. Importantly, this would also identify where these projection neurons are located within the dorsal horn, the proportion within sensory‐processing laminae I‐LV, the DGC, and in autonomic nuclei.

We observed a sex difference in the amount of CRD neuronal activation evoked in the LC, with noxious CRD evoking an increase in pERK‐IR neurons relative to no CRD controls in female mice but not male mice. We also observed a heightened level of baseline neuronal activity in the BR of female mice. The underlying mechanism of these sex differences warrants further investigation, as they have implications to sex‐differences in colorectal nociception and affective defecation reflexes in health and disease. They are possibly a factor of the sex differences evident in the sensitivity profiles of colorectal pelvic afferent fibers that have been linked to differences in visceral sensitivity and anxiety behaviors in female mice (Bayrer et al. 2023). Sex differences in the sensitivity of the LC and BR to incoming information may also be a factor. There is evidence for sex differences in the evoked activity of the LC being mediated by increased sensitivity to corticotropin‐releasing factor in females (Bangasser et al. 2016). The LC is one of the main stress‐responsive nuclei of the brain, and this hypersensitivity is believed to contribute to the greater vulnerability of females to stress‐related disorders (Bangasser et al. 2016). Aligning with this, chemogenetic activation of LC‐RVM circuits evokes both anxiety‐like behaviors and colorectal hypersensitivity (Kong et al. 2023). While inhibition of these circuits alleviates anxiety and colorectal hypersensitivity induced by colitis and stressors. As such, these sex differences may be a contributing factor to the predominately female nature of irritable bowel syndrome.

DRG removal did not influence CRD‐evoked neuronal activation in the NTS or DMV. This provides insight into the influence the vagal pathways have on colorectal sensory processing within the NTS over that of spinosolitary tracts. In agreement, vagal denervation, rather than spinalization, has previously been shown to significantly attenuate neuronal activation within the NTS, in addition to the rVLM, evoked from the proximal colon (Monnikes et al. 2003). We observed very few EGFP‐H129+ cells within the NTS96 to 1200 h after colorectal injection. However, we observed pERK‐IR neurons in the NTS. The length of the balloon used for CRD would activate afferent fibers within the proximal and distal colon, whilst the HSV1‐H129‐EGFP injections were localized to the distal colon. The vagal afferent innervation of the colorectum occurs in a gradient. Anterograde tracing from the nodose ganglia in rats and mice shows vagal afferent nerve endings within the proximal colon that become sparse or are absent in the distal colon through to the rectum (Wang and Powley 2000; Spencer et al. 2024). Aligning with this, a greater proportion of neurons within the nodose ganglia are retrogradely labeled from the proximal colon compared to the distal colon (Osman et al. 2023; Wang et al. 2024). In agreement, we have demonstrated that fibers in the NTS retrogradely labeled from the proximal colon are more abundant than fibers labeled from the distal colon (Wang et al. 2024). Moreover, fibers labeled from the proximal colon arborize extensively within the NTS and are also present within the DMV, which may be reflected in the abundance of CRD‐evoked pERK‐IR neurons in these locations. We have found that a proportion (13%) of CRD‐activated neurons in the NTS project to the lPbN (Wang et al. 2024). Based on the findings from this study, this is a minor contribution to the overall CRD‐evoked neuronal activation in the lPbN relative to spinal input. Unlike the NTS, we observed EGFP‐H129+ cells in abundance within the DMV. This is a consequence of retrograde labeling from DMV efferent projections that have been shown within the myenteric ganglia extending into the distal colon (Berthoud et al. 1991; Tao et al. 2021). Aligning with this, we have shown that DMV neurons are retrogradely labeled from the proximal and distal colon (Wang et al. 2024).

Collectively, this study identified the spinal cord and brainstem structures involved in the relay and processing of colorectal sensory‐motor information in the mouse. This study also identified that the two spinal afferent pathways innervating the colorectum relay into common and unique brainstem networks relevant to ascending sensory relay, descending pain modulation, and autonomic responses. Such new insights into the brain circuitry that splanchnic and pelvic colorectal afferents relay into aid interpretation of their respective roles in affecting appropriate sensory‐motor responses. These findings also have implications for understanding how peripheral changes within either of these afferent pathways, such as that evident during and post‐colitis, may lead to altered brain processing supporting colorectal hypersensitivity, visceral pain‐induced stress, and dysfunctional motility.

## Author Contributions

**QingQing Wang:** data curation, formal analysis, visualization, investigation, writing – review and editing. **Alice E. McGovern:** data curation, visualization, writing – review and editing, methodology, investigation. **Melinda Kyloh:** methodology, investigation. **Grigori Rychkov:** conceptualization, writing – review and editing, supervision. **Nick J. Spencer:** conceptualization, methodology, writing – review and editing, investigation. **Stuart B. Mazzone:** conceptualization, formal analysis, methodology, investigation, writing – review and editing, funding acquisition, resources. **Stuart M. Brierley:** conceptualization, writing – original draft, methodology, investigation, supervision, writing – review and editing, funding acquisition, resources. **Andrea M. Harrington:** conceptualization, data curation, formal analysis, visualization, writing – original draft, methodology, investigation, supervision, project administration, writing – review and editing, validation, funding acquisition, resources.

## Disclosure

Infrastructure: Instruments and expertise of Microscopy Australia (ROR: 042mm0k03) at Adelaide Microscopy, University of Adelaide, enabled by NCRIS, the university, and the South Australian government support.

## Conflicts of Interest

The authors declare no conflicts of interest as the research was conducted in the absence of any commercial or financial relationships that could be constructed as a potential conflict of interest. Parts of this work have appeared in presentations at the Australian Neuroscience Society annual meeting 2019 and Society for Neuroscience: Global Connectome Virtual Conference 2021.

## Peer Review

The peer review history for this article is available at https://www.webofscience.com/api/gateway/wos/peer‐review/10.1111/jnc.70211.

## Supporting information


**Data S1:** jnc70211‐sup‐0001‐FigureS1‐S3‐TableS1.pdf.

## Data Availability

The data that support the findings of this study are available from the corresponding author upon reasonable request. A preprint of this article was posted on BioRxiv on February 25, 2025; https://www.biorxiv.org/content/10.1101/2025.02.24.639793v1.
