# Supplementary material for: Splanchnic and Pelvic Spinal Afferent Pathways Relay Sensory Information From the Mouse Colorectum Into Distinct Brainstem Circuits
Source: J Neurochem. 2025 Sep 1;169(9):e70211. doi: 10.1111/jnc.70211 (PMC12401814; doi:10.1111/jnc.70211)
Supplement: Supplementary file 1 — Data S1: jnc70211‐sup‐0001‐FigureS1‐S3‐TableS1.pdf. [file JNC-169-0-s001.pdf]

SUPPLEMENTARY DATA

Splanchnic and pelvic spinal afferent pathways relay sensory information from the mouse colorectum into distinct brainstem circuits.

QingQing Wang[1, 2], Alice E. McGovern[3], Melinda Kyloh[4], Grigori Rychkov[5], Nick J. Spencer[4], Stuart B. Mazzone[3], Stuart M. Brierley[1, 5], Andrea M. Harrington*[1, 2, 5].

[1] Visceral Pain Research Group, Hopwood Centre for Neurobiology, Lifelong Health Theme, South Australian Health and Medical Research Institute (SAHMRI), North Terrace, Adelaide, South Australia, AUSTRALIA.

[2] College of Medicine and Public Health, Flinders Health and Medical Research Institute, Flinders University, Bedford Park, South Australia, 5042, AUSTRALIA.

[3] Department of Anatomy and Physiology, The University of Melbourne, Parkville, VIC, Australia

[4] Visceral Neurophysiology Laboratory, College of Medicine and Public Health, Flinders Health and Medical Research Institute, Flinders University, Bedford Park, SA, AUSTRALIA.

[5] School of Biomedicine, Faculty of Health and Medical Sciences, University of Adelaide, Adelaide, SA, AUSTRALIA.

*** Correspondence:**
Dr. Andrea M. Harrington
andrea.harrington@adelaide.edu.au

Keywords: Distal colon, rectum, spinal cord, visceral afferent, brainstem and pain.

Abbreviations: 4V = Fourth ventricle, 5-HT = serotonin, AP = area postrema, br = bregma, BR = Barrington's nucleus, cVLM = caudal ventrolateral medulla, CRD = colorectal distension**,** DVC = dorsal vagal complex, DGC= dorsal grey commissure, DMV = dorsal motor nucleus of the vagus, DRG = dorsal root ganglia, DRN = dorsal raphe nuclei**,** Gi =

gigantocellular reticular nucleus, HSV1 H129-EGFP = herpes simplex virus 1, H129 strain, expressing green fluorescent protein, IRt = intermediate reticular nucleus, ici= intercalated nuclei, IML= intermediolateral nuclei, IR = immunoreactive, KF = Kölliker-Fuse nucleus, LI= lamina I, LII= lamina II, LIII= lamina III, LIV= lamina 4, LV= lamina 5, LSN= lateral spinal nucleus, lPbN = lateral parabrachial nuclei, LPgi = lateral paragigantocellular nucleus, LRt = lateral reticular nucleus, LPGi = lateral paragigantocellular nucleus, LS= lumbosacral, LC = locus coeruleus, mPbN = medial parabrachial nuclei, NA = nucleus ambiguus, NTS = nucleus of the solitary tract, pERK = phosphorylated map kinase ERK1/2, PAG = periaqueductal gray, RMg = raphe magnus nucleus, RPa = raphe pallidus nucleus, rVLM = rostroventrolateral reticular nucleus, ROb= raphe obscurus nucleus, RVM = rostral ventromedial medulla, rVLM = rostral ventrolateral medulla, SPN= sacral parasympathetic nuclei scp = superior cerebellar peduncle, SubC = sub coeruleus, and TL= thoracolumbar.

Supplementary Figure 1: HSV-1 H129-EGFP in the distal colon

A) Schematic showing the injection sites of HSV-1 H129-EGFP (green) at multiple sites into the subserosa-musculature wall of the mouse distal colon and rectum (colorectum), covering the region 0.2 cm below the pelvic bone through to 1 cm proximal to the pelvic bone.

Representative photomicrographs of colorectum in cross-section distal and proximal to the injection site of H129-EGFP+ **B)** 24-, **C)** 48-, **D)** 72-, **E)** 96- and **F)** 120 hours after HSV-1 H129-EGFP injection. Scale bar = 200μm.

SUPP. FIGURE 1

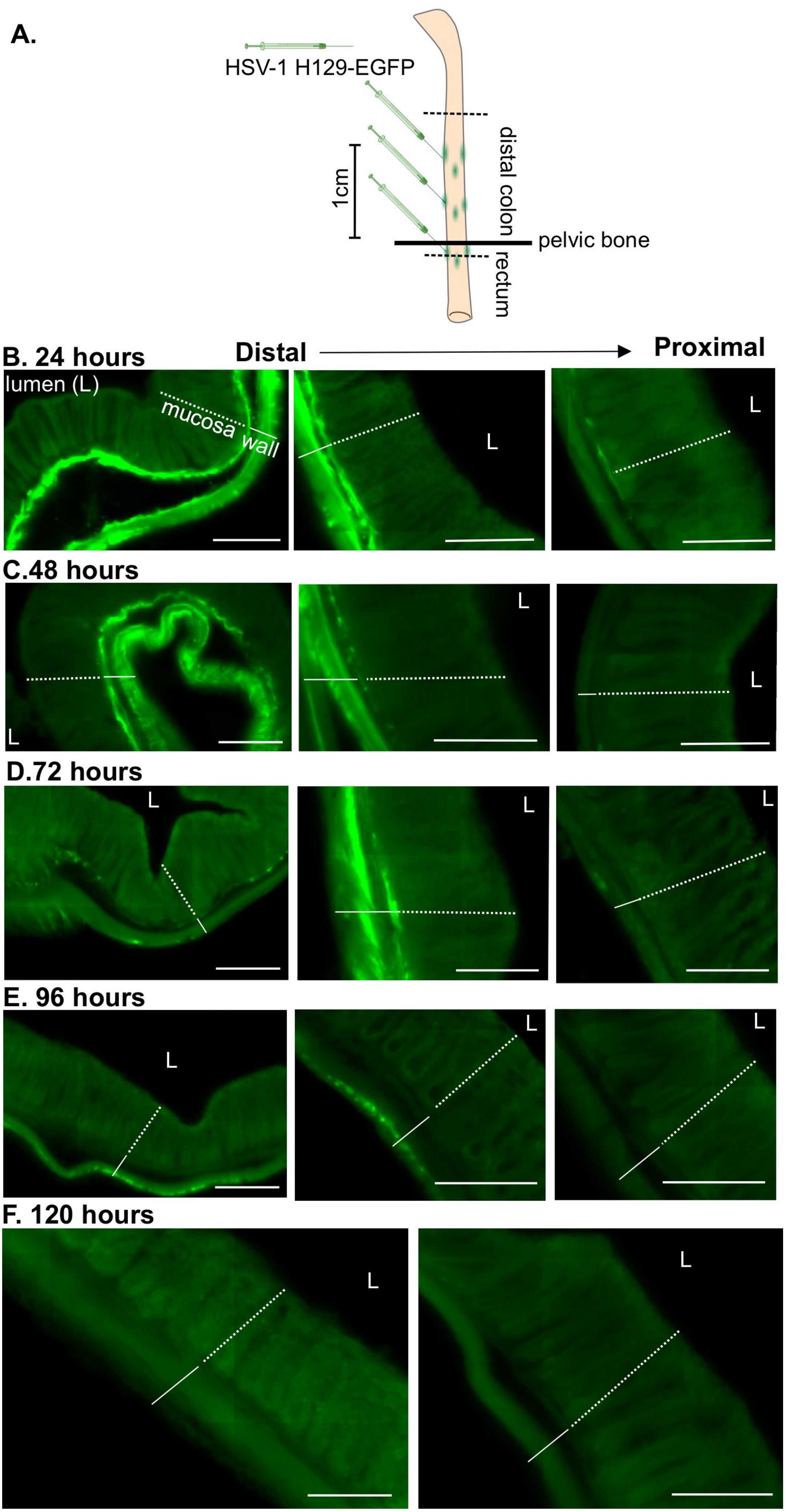

Supplementary Figure 3: HSV-1 H129-EGFP in the medulla, pons and midbrain 72-120 hours post-inoculation.

A-C) Photomicrographs showing the distribution of H129-EGFP+ cells (green) in the **A)** caudal ventrolateral medulla (cVLM), **B)** rostral ventromedial medulla (RVM) and **C)** Barrington's nucleus (BR) and the locus coeruleus (LC) in tissue collected 72-, 96-, 120 hours post-HSV-1 H129-EGFP colorectal inoculation. Scale bars: 100 µm.

D and E) Photomicrographs showing the distribution of H129-EGFP+ cells (green) in the lateral parabrachial nucleus (lPbN) and the **A)** lateral (lPAG) and ventrolateral (vlPAG) sub-regions of the periaqueductal gray 96 and 120 hours post-inoculation of HSV-1 H129-EGFP within the colorectal wall. Scale bars: 100 µm.

Abbreviations: LRt = lateral reticular nucleus, RMg = raphe magnus nucleus.

SUPP. FIGURE 2

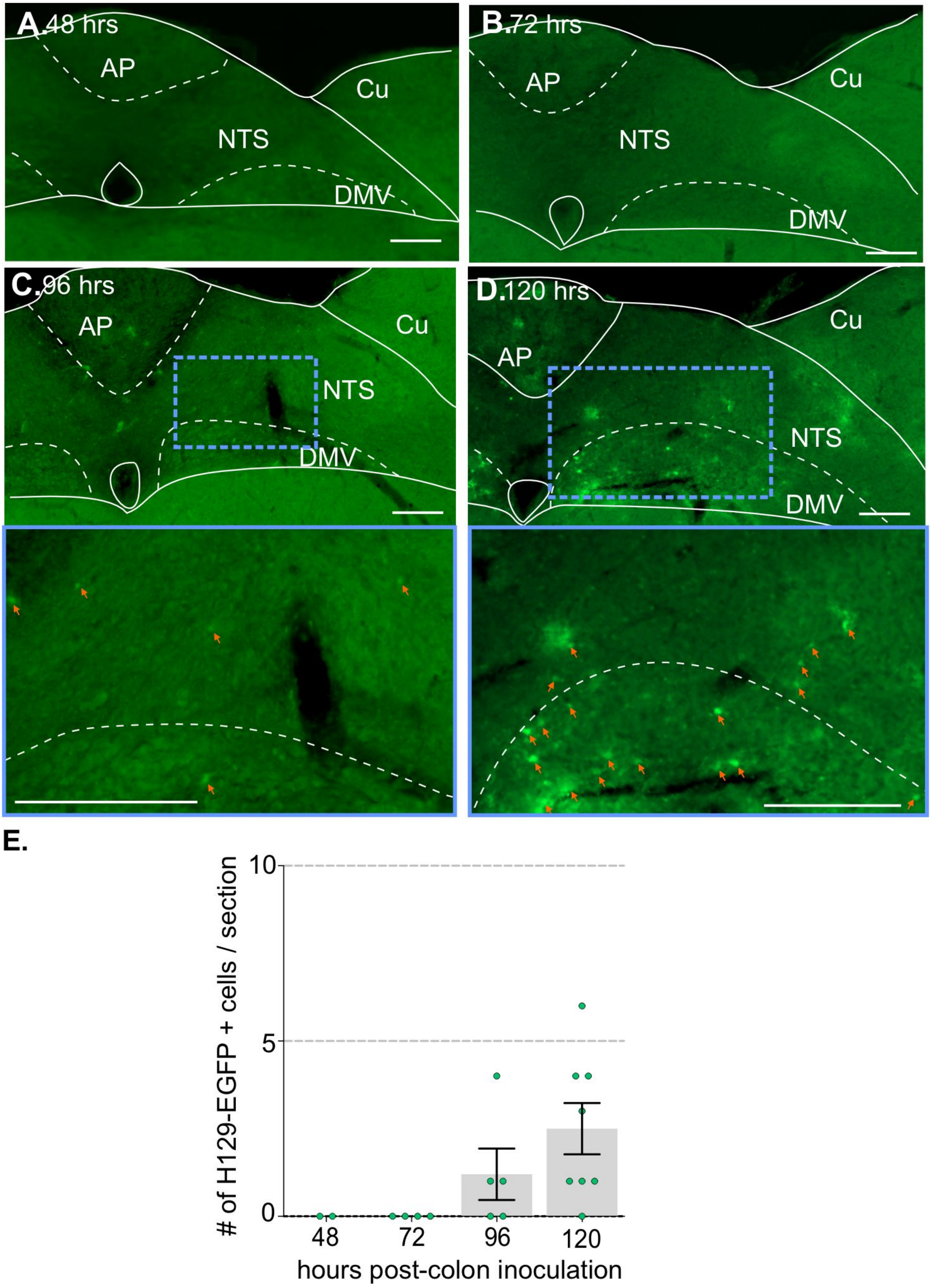

Supplementary Figure 2: HSV-1 H129-EGFP in the dorsal vagal complex.

Photomicrographs of the dorsal vagal complex nucleus of the solitary tract (NTS) and dorsal motor nucleus of the vagus (DMV) **A)** 48-, **B)** 72-, **C)** 96- and **D)** 120 hours post-inoculation of HSV-1 H129-EGFP within the colorectal wall. Scale bars: 500 μm. **E)** Quantification of the mean number of H-129 EGFP+ cells within sections of nucleus of the solitary tract (NTS) compared between tissue collected 48-, 72-, 96-, 120 hours post-HSV-1 H129-EGFP colorectal inoculation. Data compared using One-Way ANOVA (parametric data) with Bonferroni multiple comparison tests. Data points represent the mean number of H129-EGFP+ cells/section of individual mice, gained from 2-3 sections/mouse collected 48 hours (N=2 1M:1F), 72 hours (N= 4 mice 2F:2M), 96 hours (N= 5 mice 3F:2M) and 120 hours (N= 8 mice 7F:1M) post-inoculation. Error bars represent the standard error of the mean.

Abbreviations: AP = Area postrema, Cu = cuneate nuclei.

SUPP. FIGURE 3

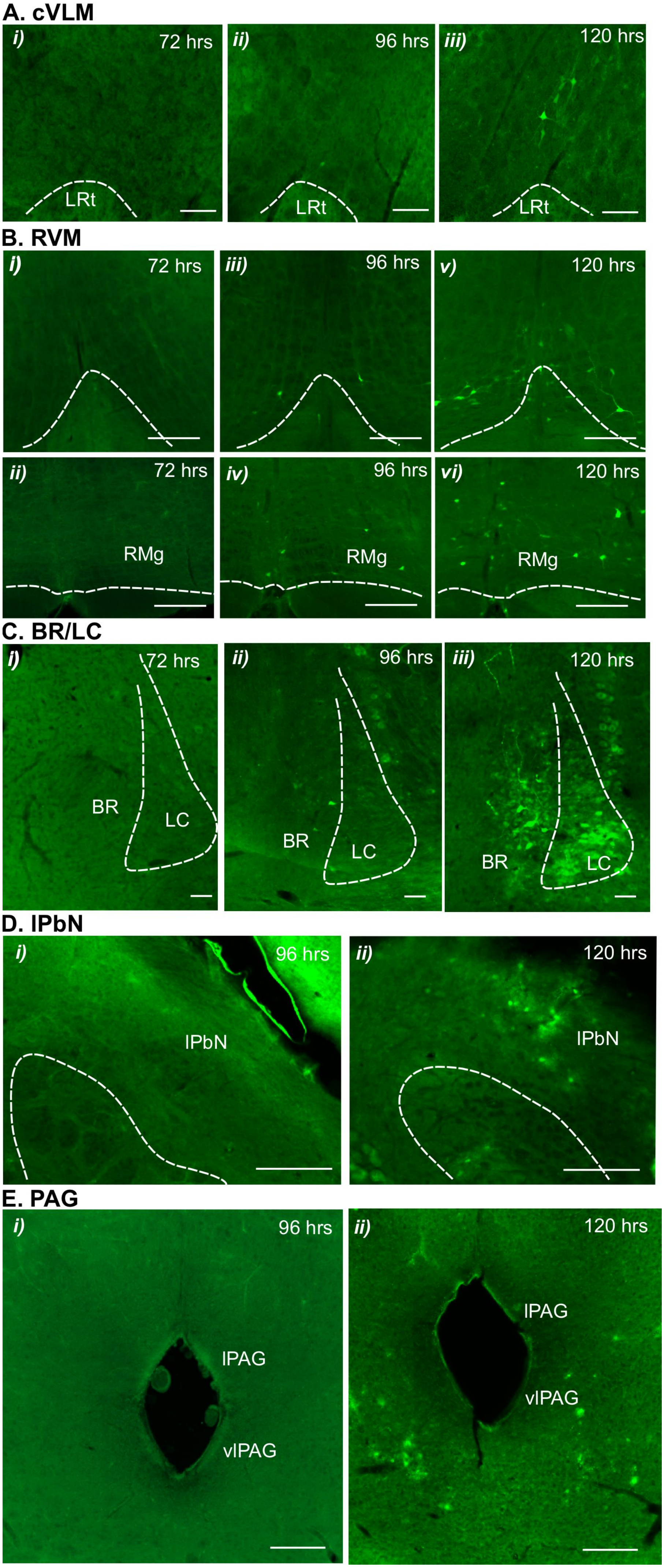

Supplementary Table 1: Table of analysis.

A full statistical report of all data sets ordered by Figure appearance, inclusive of ANOVA (degrees of freedom (df), F value, actual P value), t-test include (df, t value, actual P value) and Kruskal-Wallis test, please report the (H statistic, df, and P value).

**Figure**	**comparison**	**test**	**P value**	**df**	**H stats**	**F value**	**T value**
**1**	H129 EGFP 48 hours	Ordinary one-way ANOVA	0.02	14	n/a	4.6	n/a
**1**	H129 EGFP 72 hours	Ordinary one-way ANOVA	0.0004	15	n/a	13.38	n/a
**1**	H129 EGFP 96 hours TL spinal cord vs all regions	Ordinary one-way ANOVA	0.0009	40	n/a	4.51	n/a
**1**	H129 EGFP 96 hours LS spinal cord vs all regions	Ordinary one-way ANOVA	<0.0001	51	n/a	5.3	n/a
**1**	H129 EGFP 120 hours TL spinal cord vs all regions	Ordinary one-way ANOVA	<0.0001	81	n/a	4.54	n/a
**1**	H129 EGFP 120 hours LS spinal cord vs all regions	Ordinary one-way ANOVA	0.0001	70	n/a	4.5	n/a
**2Bvi**	H129 EGFP in TL DRG 24-120 hours	Kruskal-Wallis test	0.015	125	12.27	n/a	n/a
**2Cvi**	H129 EGFP in LS DRG 24-120 hours	Kruskal-Wallis test	<0.0001	291	66.17	n/a	n/a
**3Bvi**	H129 EGFP in TL DH 24-120 hours	Ordinary one-way ANOVA	0.014	22	n/a	4.18	n/a
**3Cvi**	H129 EGFP in LS DH 24-120 hours	Ordinary one-way ANOVA	0.0089	22	n/a	4.70	n/a
**4B**	H129 EGFP in LI DH 72-120 hours	Ordinary one-way ANOVA	0.02	16	n/a	4.90	n/a
**4B**	H129 EGFP in LII-V DH 72-120 hours	Ordinary one-way ANOVA	0.02	16	n/a	5.00	n/a
**4B**	H129 EGFP in DGC DH 72-120 hours	Ordinary one-way ANOVA	0.39	16	n/a	0.98	n/a
**4B**	H129 EGFP in IML DH 72-120 hours	Ordinary one-way ANOVA	0.22	16	n/a	1.63	n/a
**5B**	H129 EGFP in LI DH 72-120 hours	Ordinary one-way ANOVA	<0.0001	16	n/a	21.18	n/a
**5B**	H129 EGFP in LII-V DH 72-120 hours	Ordinary one-way ANOVA	0.007	16	n/a	7.031	n/a
**5B**	H129 EGFP in DGC 72-120 hours	Ordinary one-way ANOVA	0.019	15	n/a	5.42	n/a
**5B**	H129 EGFP in SPN 72-120 hours	Ordinary one-way ANOVA	0.018	16	n/a	5.39	n/a
**6B**	H129 EGFP in NTS 72-120 hours	Ordinary one-way ANOVA	0.08	16	n/a	2.99	n/a
**6B**	H129 EGFP in DMV 72-120 hours	Ordinary one-way ANOVA	0.145	16	n/a	2.21	n/a
**6B**	H129 EGFP in cVLM 72-120 hours	Ordinary one-way ANOVA	0.0004	16	n/a	14.17	n/a
**6B**	H129 EGFP in RVM 72-120 hours	Ordinary one-way ANOVA	0.0011	16	n/a	11.55	n/a
**7B**	H129 EGFP in BR 72-120 hours	Ordinary one-way ANOVA	0.0010	16	n/a	11.90	n/a
**7B**	H129 EGFP in LC 72-120 hours	Ordinary one-way ANOVA	0.0002	16	n/a	17.34	n/a

**7B**	H129 EGFP in lPbN 72-120 hours	Ordinary one-way ANOVA	0.0002	16	n/a	16.66	n/a
**8B**	H129 EGFP in PAG 72-120 hours	Ordinary one-way ANOVA	0.012	13	n/a	6.65	n/a
**9Ciii**	pERK-IR in DMV female 0mmHg vs 80mmHg	Unpaired t-test	0.24	8	n/a	n/a	1.26
**9Ciii**	pERK-IR in DMV male 0mmHg vs 80mmHg	Unpaired t-test	0.0003	7	n/a	n/a	6.55
**9Ciii**	pERK-IR in DMV male 80mmHg vs female 80mmHg	Unpaired t-test	0.009	7	n/a	n/a	3.5
**9Civ**	pERK-IR in NTS female 0mmHg vs 80mmHg	Unpaired t-test	<0.0001	7	n/a	n/a	17.36
**9Civ**	pERK-IR in NTS male 0mmHg vs 80mmHg	Unpaired t-test	0.0003	8	n/a	n/a	5.9
**9Div**	pERK-IR in cVLM female 0mmHg vs 80mmHg	Unpaired t-test	0.002	8	n/a	n/a	4.32
**9Div**	pERK-IR in cVLM male 0mmHg vs 80mmHg	Unpaired t-test	0.005	8	n/a	n/a	3.807
**9Eiii**	pERK-IR in RVM female 0mmHg vs 80mmHg	Unpaired t-test	0.01	8	n/a	n/a	3.3
**9Eiii**	pERK-IR in RVM male 0mmHg vs 80mmHg	Unpaired t-test	0.008	8	n/a	n/a	3.45
**10Civ**	pERK-IR in BR female 0mmHg vs 80mmHg	Unpaired t-test	0.12	8	n/a	n/a	1.69
**10Civ**	pERK-IR in BR male 0mmHg vs 80mmHg	Unpaired t-test	0.0003	7	n/a	n/a	6.7
**10Civ**	pERK-IR in BR male 0mmHg vs female 0mmHg	Unpaired t-test	0.03	7	n/a	n/a	2.64
**10Cv**	pERK-IR in LC female 0mmHg vs 80mmHg	Unpaired t-test	0.03	8	n/a	n/a	2.63
**10Cv**	pERK-IR in LC male 0mmHg vs 80mmHg	Unpaired t-test	0.35	7	n/a	n/a	0.98
**10Cv**	pERK-IR in LC male 80mmHg vs female 80mmHg	Unpaired t-test	0.02	8	n/a	n/a	2.76
**10Div**	pERK-IR in lPbN female 0mmHg vs 80mmHg	Unpaired t-test	0.0001	8	n/a	n/a	7.068
**10Div**	pERK-IR in lPbN male 0mmHg vs 80mmHg	Unpaired t-test	0.0002	8	n/a	n/a	6.48
**11Ciii**	pERK-IR in PAG female 0mmHg vs 80mmHg	Unpaired t-test	0.02	8	n/a	n/a	2.814
**11Ciii**	pERK-IR in PAG male 0mmHg vs 80mmHg	Unpaired t-test	0.001	8	n/a	n/a	4.59
**13Biv**	pERK-IR in T13-L1 lesion vs sham ALL	Ordinary one-way ANOVA	0.0004	14	n/a	16.28	n/a
**13Biv**	pERK-IR in T13-L1 lesion vs sham LI	Ordinary one-way ANOVA	0.0007	14	n/a	14.37	n/a
**13Biv**	pERK-IR in T13-L1 lesion vs sham LII-V	Ordinary one-way ANOVA	0.07	14	n/a	3.2	n/a
**13Biv**	pERK-IR in T13-L1 lesion vs sham DGC	Ordinary one-way ANOVA	0.013	14	n/a	6.3	n/a
**13Biv**	pERK-IR in T13-L1 lesion vs sham IML	Ordinary one-way ANOVA	0.54	14	n/a	0.63	n/a
**13Civ**	pERK-IR in L6-S1 lesion vs sham ALL	Ordinary one-way ANOVA	0.01	14	n/a	6.82	n/a
**13Civ**	pERK-IR in L6-S1 lesion vs sham LI	Ordinary one-way ANOVA	0.02	14	n/a	5.3	n/a
**13Civ**	pERK-IR in L6-S1 lesion vs sham LII-V	Ordinary one-way ANOVA	0.01	14	n/a	6.14	n/a
**13Civ**	pERK-IR in L6-S1 lesion vs sham DGC	Ordinary one-way ANOVA	0.017	14	n/a	5.8	n/a
**13Civ**	pERK-IR in L6-S1 lesion vs sham SPN	Ordinary one-way ANOVA	0.22	14	n/a	1.70	n/a

**14Aiv**	pERK-IR in DMV lesion vs sham	Ordinary one-way ANOVA	0.5	14	n/a	0.72	n/a
**14Aiv**	pERK-IR in NTS lesion vs sham	Ordinary one-way ANOVA	0.6	13	n/a	0.45	n/a
**14Biv**	pERK-IR in cVLM lesion vs sham	Ordinary one-way ANOVA	0.01	14	n/a	5.94	n/a
**14Civ**	pERK-IR in RVM lesion vs sham	Ordinary one-way ANOVA	0.007	13	n/a	8	n/a
**15Aiv**	pERK-IR in BR lesion vs sham	Ordinary one-way ANOVA	0.054	14	n/a	3.7	n/a
**15Aiv**	pERK-IR in LC lesion vs sham	Ordinary one-way ANOVA	0.017	14	n/a	5.79	n/a
**15Biv**	pERK-IR in lPbN lesion vs sham	Ordinary one-way ANOVA	0.002	14	n/a	10.60	n/a
**15Civ**	pERK-IR in PAG lesion vs sham	Ordinary one-way ANOVA	0.022	12	n/a	5.64	n/a
